# Intervisit Reproducibility of Foveal Cone Density Metrics

**DOI:** 10.1167/tvst.13.6.18

**Published:** 2024-06-24

**Authors:** Iniya Adhan, Emma Warr, Jenna Grieshop, Joseph Kreis, Danica Nikezic, Ashleigh Walesa, Katherine Hemsworth, Robert F. Cooper, Joseph Carroll

**Affiliations:** 1 Department of Ophthalmology & Visual Sciences, Medical College of Wisconsin, Milwaukee, WI, USA; 2 Joint Department of Biomedical Engineering, Marquette University and Medical College of Wisconsin, Milwaukee, WI, USA; 3 Department of Cell Biology, Neurobiology & Anatomy, Medical College of Wisconsin, Milwaukee, WI, USA

**Keywords:** adaptive optics, cone density, photoreceptors

## Abstract

**Purpose:**

To assess longitudinal reproducibility of metrics of foveal density (peak cone density [PCD], cone density centroid [CDC], and 80th percentile centroid area) in participants with normal vision.

**Methods:**

Participants (*n* = 19; five male and 14 female) were imaged at two time points (average interval of 3.2 years) using an adaptive optics scanning light ophthalmoscope (AOSLO). Foveally centered regions of interest (ROIs) were extracted from AOSLO montages. Cone coordinate matrices were semiautomatically derived for each ROI, and cone mosaic metrics were calculated.

**Results:**

On average, there were no significant changes in cone mosaic metrics between visits. The average ± SD PCD was 187,000 ± 20,000 cones/mm^2^ and 189,000 ± 21,700 cones/mm^2^ for visits 1 and 2, respectively (*P* = 0.52). The average ± SD density at the CDC was 183,000 ± 19,000 cones/mm^2^ and 184,000 ± 20,800 cones/mm^2^ for visits 1 and 2, respectively (*P* = 0.78). The average ± SD 80th percentile isodensity contour area was 15,400 ± 1800 µm^2^ and 15,600 ± 1910 µm^2^ for visits 1 and 2, respectively (*P* = 0.57).

**Conclusions:**

Foveal cone mosaic density metrics were highly reproducible in the cohort examined here, although further study is required in more diverse populations.

**Translational Relevance:**

Determination of the normative longitudinal changes in foveal cone topography is key for evaluating longitudinal measures of foveal cone topography in patients with progressive retinal dystrophies.

## Introduction

Adaptive optics scanning light ophthalmoscopy (AOSLO) allows non-invasive visualization of the photoreceptor mosaic in the living human retina.^1^ Since publication in 2007 of the first clinical report on the use of AOSLO to characterize photoreceptor structure in patients with retinitis pigmentosa or cone–rod dystrophy,^2^ there have been hundreds of studies using AOSLO (or other AO-based imaging modalities) to examine photoreceptor structure in a wide range of patient populations.^3^^–^^8^ AOSLO images can be used to identify structural damage that is not evident on clinical images^9^^–^^11^ or to facilitate structure–function correlations to better understand disease etiology.^6^^,^^12^^–^^14^ However, the true clinical utility of photoreceptor imaging with AOSLO is reliant on the ability to extract quantitative metrics from these images.^15^ Of particular interest is the foveal cone mosaic, which underlies our high-acuity daytime vision. Although cross-sectional studies describing multiple metrics of foveal cone topography have been conducted,^16^^–^^20^ application of these metrics to longitudinal clinical studies requires a comprehensive understanding of their reliability, repeatability, and reproducibility.

Multiple studies have examined the variability of parafoveal cone metrics (i.e., measurement error, reliability, repeatability, and reproducibility) under changing experimental conditions, such as device, grader, and time. Liu et al.^21^ described both interdevice and interobserver reliability and reproducibility, with the largest contribution to variability being the participants themselves compared to variability seen between devices (2.5%–6.9%) or graders (1.3%). Good intrasession repeatability has been observed from eccentricities greater than 0.5° from the central fovea in a normative population, with repeatability coefficients as low as 2.7%.^22^^,^^23^ Jackson et al.^24^ found that intersession parafoveal densities were highly repeatable over a 2-year follow-up period when using regions of interest (ROIs) that were aligned between the time points being assessed. These studies all support the general conclusion that metrics of the parafoveal cone mosaic can be reliably and reproducibly derived from AOSLO images of the living retina.

Fewer studies have examined the foveal cone mosaic, due in part to the challenges associated with reliable visualization of the smallest central foveal cones.^25^^,^^26^ Interocular symmetry with metrics such as peak cone density (PCD), mosaic regularity, and isodensity contour area has been seen among individuals with normal vision.^16^ Intergrader repeatability was also examined by Wynne et al.,^27^ who observed an 11.7% intergrader measurement error in PCD estimates but better reproducibility of density measurements when extracting density at the cone density centroid (CDC) location. Here, we conducted a prospective study to examine longitudinal reproducibility of three commonly used metrics to describe the foveal cone mosaic (PCD, CDC, and 80th percentile isodensity contour area). These metrics were chosen because they are commonly reported by many groups when assessing AOSLO images of the cone mosaic.^28^^,^^29^ These data can serve as a reference for assessing foveal cone metrics over time in patients with inherited retinal degenerations, monitoring age-related changes in the cone mosaic,^30^ or even assessing developmental changes in the cone mosaic in younger individuals.

## Methods

### Participants

This study followed the tenets of the Declaration of Helsinki and was approved by the Medical College of Wisconsin Institutional Review Board (PRO30741). A total of 19 participants (age range at baseline: 12–64 years; 14 females and five males) were recruited for this study, all of whom were also previously imaged as part of the study by Cava et al.^16^ The average time between visits was 3.2 years (range, 2.48–4.28).

### AOSLO Imaging and Processing

The foveal cone mosaic in one eye of each participant was imaged at two time points using a confocal AOSLO. Prior to most imaging sessions, autorefraction was performed (KR-800S Autorefractor/Keratometer; Topcon Corporation, Tokyo, Japan) to estimate the base spherical correction required for AOSLO. Before AOSLO imaging, a dental impression on a bite bar was used to stabilize the head. The participants fixated at different locations so that the central foveal region was sampled at approximately 0.5° intervals. The imaging field of view spanned 1°, thus producing approximately 50% overlap between neighboring videos. Videos consisting of 150 to 200 frames were collected at each imaging location, using a 775-nm or 790-nm superluminescent diode (SLD) to illuminate the retina.

Various imaging and processing protocols were implemented depending on the resolution necessary to resolve the foveal cones of a given retina. These included using a 680-nm SLD (incident power 32.5 µW), imaging over a smaller field of view (0.5° or 0.75°), and/or using a sub-Airy disk pinhole (0.5–0.7 Airy disk diameter).^16^ Another imaging technique involved repeatedly imaging at the same foveal location at different planes of focus or at different time points of the imaging session.^31^

Raw videos were processed as previously described in Cava et al.^16^ A minimally distorted reference frame from each video was automatically chosen using a previously described algorithm,^32^ which was then used to register and average the remaining frames in the video using a strip-based registration algorithm.^32^^,^^33^ To eliminate further distortion, dewarping software was used (https://github.com/OCVL/Eye-Motion-Repair); this software is based on the method described by Bedggood and Metha.^34^ This script estimates random eye motion distortions throughout the reference frame, which are then used to calculate the median translation observed at the row of the registered images. The median translation is then used to fix the distortion of the reference frame in the equal but opposite direction.^16^^,^^35^ The result is a high signal-to-noise ratio image for each video acquired. In instances where multiple videos were collected at the same location at different time points or at different planes of focus, the videos were processed as above and then averaged using StackReg^36^ within ImageJ (National Institutes of Health, Bethesda, MD) to produce images with more uniform cone reflectance.

### Montage Generation and ROI Extraction

From these images, a montage was created using a custom MATLAB (MathWorks, Natick, MA) automontaging script (https://github.com/BrainardLab/AOAutomontaging) that overlapped processed images to create a larger montage.^37^ The montage was then imported into Photoshop CS6 (Adobe, San Jose, CA) to examine the output for any alignment errors of neighboring images, which were manually repositioned if needed. When alignment had been confirmed, layers around the region of perceived highest cone density were manually blended to create a flattened seamless foveal image. Using each participant's axial length and known system scale, the scale of each foveal image was calculated, and an ROI was cropped for analysis. For visit 1, the ROI was a 300 × 300-µm square; for visit 2, the ROI was a 500 × 500-µm square. The larger ROI for visit 2 was used to provide a greater chance of capturing the same foveal location at both visits.

### Extracting Foveal Cone Mosaic Metrics

A semiautomatic cell-marking software (Mosaic Analytics; Translational Imaging Innovations, Hickory, NC) was used to identify initial cone coordinates within each ROI (graders IA, EW, AW, or KH). A review of these coordinates was performed by an experienced grader (JC) to generate a final coordinate list for each ROI. All of the ROIs and coordinate files were then rescaled to a common scale. Density matrices were then derived using a square sampling window that varied in size to include 150 cones (with Voronoi domains fully contained within the window) at each point sampled within the ROI using a modified MATLAB script (https://github.com/AOIPLab/Metricks/releases/tag/Adhan_et_al_2024). From these matrices, we derived the location and values of the PCDs and CDCs, along with the 80th percentile isodensity contour area (area of the density matrix containing density values in the top 20% of all densities within the ROI) as described by Wynne et al.^27^ In addition, we examined the relative offset between the PCD and CDC locations.

Difference maps were generated by aligning the visit 1 and visit 2 density matrices for a given subject (to their respective CDC locations) and subtracting the overlapping region, resulting in a 300 × 300-µm map. Horizontal and vertical cross-sections were then extracted through the CDC location of each subject's difference map and averaged in 5-µm sampling windows. All overlapping sampling windows between subjects were averaged to create composite horizontal and vertical difference profiles.

### Statistics

Summary statistics for each density metric were calculated using both linear (cones/mm^2^) and angular (cones/deg^2^) units. Raw values were rounded to three significant digits, representing the level of uncertainty in our image scale measure. A Shapiro–Wilk normality test was used to assess the normality of intervisit differences for linear and angular densities of the PCDs and CDCs and for the 80th percentile isodensity contour areas between the two visits for each participant (Prism 9.0.0; GraphPad Software, Boston, MA). This is important, because Bland–Altman analysis assumes that the differences are normally distributed.^38^ Intervisit agreement for all metrics was assessed with a Bland–Altman analysis and Pearson's correlation analyses.

## Results

The mean ± SD time elapsed between the two visits was 3.20 ± 0.51 years. The intervisit PCD differences and density differences at the CDC location were all normally distributed using both linear units (*P* = 0.79 and *P* = 0.98, respectively) and angular units (*P* = 0.97 and *P* = 0.89, respectively). The 80th percentile isodensity contour area differences were also normally distributed (*P* = 0.41). The PCD–CDC offset differences were also normally distributed (*P* = 0.65). The mean ± SD axial length at visit 1 was 23.91 ± 0.96 mm. See Supplementary Table S1 for complete participant demographics. Individual ROIs and cone coordinate files are provided in Supplementary Datafile S1.

### Intervisit Reproducibility of PCD

**Figure 1.**
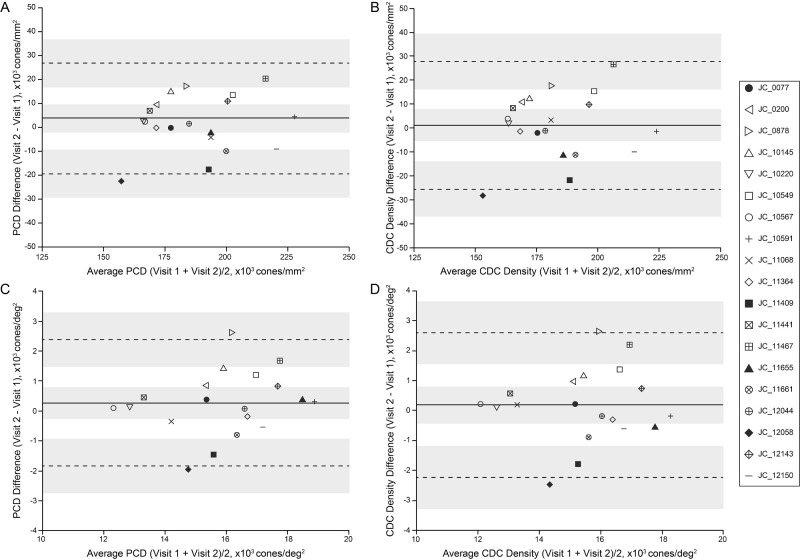
Intervisit agreement of PCD and CDC density. (**A**) We observed good agreement between visit 1 and visit 2 PCD values using linear units. The *solid line* represents the mean bias of 1740 cones/mm^2^, and the *dashed lines* represent the upper (24,000 cones/mm^2^) and lower (−20,600 cones/mm^2^) limits of agreement. (**B**) Good agreement was also observed for PCD values using angular units. The *solid line* represents the mean bias of 247 cones/deg^2^, and the *dashed lines* represent the upper (2370 cones/deg^2^) and lower (−1870 cones/deg^2^) limits of agreement. (**C**) Similar results were seen when assessing density at the CDC. We observed good agreement between visit 1 and visit 2 CDC density values using linear units. The *solid line* represents the mean bias of 895 cones/mm^2^, and the *dashed lines* represent the upper (27,700 cones/mm^2^) and lower (−25,900 cones/mm^2^) limits of agreement. (**D**) Similar agreement was seen for CDC density values using angular units. The *solid line* represents the mean bias of 163 cones/deg^2^, and the *dashed lines* represent the upper (2590 cones/deg^2^) and lower (−2260 cones/deg^2^) limits of agreement. *Shaded regions* represent the 95% CI about the mean bias and the limits of agreement.^38^ Each individual is indicated by a different symbol; the four *filled symbols* represent individuals included in Figure 3 and Supplementary Figure S1.

Figure 1 summarizes the results for PCD density (see Supplementary Table S1 for participant-level data). The mean ± SD PCD value across all participants at visit 1 was 187,000 ± 20,000 cones/mm^2^ (15,800 ± 1790 cones/deg^2^), and at visit 2 it was 189,000 ± 21,700 cones/mm^2^ (16,100 ± 2020 cones/deg^2^). The smallest intervisit percentage change in linear density was 0.56%, and the largest was 13.6%. Eleven out of 19 participants had a PCD linear density difference of less than 5%. Angular density percentage change in PCD ranged from 0% to 17.4%, with 12 out of 19 participants having a PCD angular density difference of less than 5% (all 11 participants who had less than 5% change in PCD linear densities also had less than 5% change in the PCD angular density). The PCD did not differ significantly between visits with regard to linear density (*t* = 0.66; *df* = 18; *P* = 0.52, paired *t*-test) or angular density (*t* = 0.99; *df* = 18; *P* = 0.33, paired *t*-test). There was strong correlation between visit 1 and visit 2 values with regard to linear density (Pearson correlation *r* *=* 0.85; 95% confidence interval [CI], 0.65–0.94; *P* *<* 0.0001) and angular density (Pearson correlation *r* *=* 0.85; 95% CI, 0.64–0.94; *P* *<*0.0001). Bland–Altman analysis of linear (Fig. 1A) and angular (Fig. 1B) PCD showed good agreement between visits as the 95% CI of the mean bias included zero; for linear density, the mean bias was 1740 cones/mm^2^ (95% CI, −3770 to 7240 cones/mm^2^), and, for angular density, the mean bias was 247 cones/deg^2^ (95% CI, −274 to 769 cones/deg^2^). There was no significant correlation between the intervisit interval time and absolute differences in PCD for linear density (Pearson correlation *r* *=* −0.21; 95% CI, −0.61 to 0.27; *P* *=* 0.38) or angular density (Pearson correlation *r* *=* −0.12; 95% CI, −0.55 to 0.35; *P* *=* 0.61).

### Intervisit Reproducibility of Density at the CDC Location

Figure 1 summarizes results for CDC density (see Supplementary Table S1 for participant-level data). The mean ± SD density value at the CDC location across all participants at visit 1 was 183,000 ± 19,000 cones/mm^2^ (15,400 ± 1800 cones/deg^2^), and at visit 2 it was 184,000 ± 20,800 cones/mm^2^ (15,600 ± 1910 cones/deg^2^). The CDC between visits did not significantly differ with regard to linear density (*t* = 0.28; *df* = 18; *P* = 0.78, paired *t*-test) or angular density (*t* = 0.58; *df* = 18; *P* = 0.57, paired *t*-test). There was a strong correlation between CDC values from the two visits with regard to linear density (Pearson correlation *r* *=* 0.77; 95% CI, 0.48–0.91; *P <* 0.0001) and angular density (Pearson correlation *r* *=* 0.78; 95% CI, 0.50–0.91; *P <* 0.0001). The smallest intervisit percentage change in linear density at the CDC was 0.59%, and the largest was 16.8%. Ten out of 19 participants had a difference in CDC linear density of less than 5%. Angular density percentage changes in the CDC ranged from 0.79% to 18.5%, with 11 out of 19 participants having a difference in CDC angular density of less than 5% (all 10 participants that had less than 5% change in linear CDC densities also had less than 5% change in the angular CDC density). Bland–Altman analysis of linear (Fig. 1C) and angular (Fig. 1D) density at the CCD showed good agreement between visits, as the 95% CI of the mean bias included zero; for linear density, the mean bias was 895 cones/mm^2^ (95% CI, −5700 to 7496 cones/mm^2^) and, for angular density, the mean bias was 163 cones/deg^2^ (95% CI, −433 to 760 cones/deg^2^). There was no significant correlation between the intervisit interval time and absolute differences in density at the CDC with regard to linear density (Pearson correlation *r =* −0.24; 95% CI, −0.63 to 0.24; *P* = 0.32) or angular density (Pearson correlation *r* *=* −0.16; 95% CI, −0.57 to 0.32; *P* = 0.51).

### PCD and CDC Comparisons

We found that the PCD and CDC showed similar intervisit stability. Compared to the PCD changes between visits (4.8% for linear density and 5.2% for angular density), the CDC density values showed a similar magnitude (5.8% for linear density and 5.9% for angular density). However, as expected, the absolute density values at the PCD were significantly higher than density estimates extracted at the CDC location; for linear density the mean visit 1 difference was 4740 cones/mm^2^ (*t* = 5.48; *df* = 18; *P* < 0.0001, paired *t*-test), and, for angular density, the mean visit 1 difference was 389 cones/deg^2^ (*t* = 6.01; *df* = 18; *P* < 0.0001, paired *t*-test). When comparing the spatial offset between the location of the PCD and CDC, there was no significant difference observed between visits (*t* = 1.47; *df* = 18; *P* = 0.16, paired *t*-test). The mean ± SD PCD–CDC offsets were 12.4 ± 7.03 µm at visit 1 and 14.6 ± 7.72 µm at visit 2 (see Supplementary Table S1). There was no significant correlation between the intervisit interval time and absolute difference in the PCD–CDC offset (Pearson correlation *r* *=* −0.37; 95% CI, −0.71 to 0.10; *P* = 0.11).

### Intervisit Reproducibility of the 80th Percentile Isodensity Contour Area

**Figure 2.**
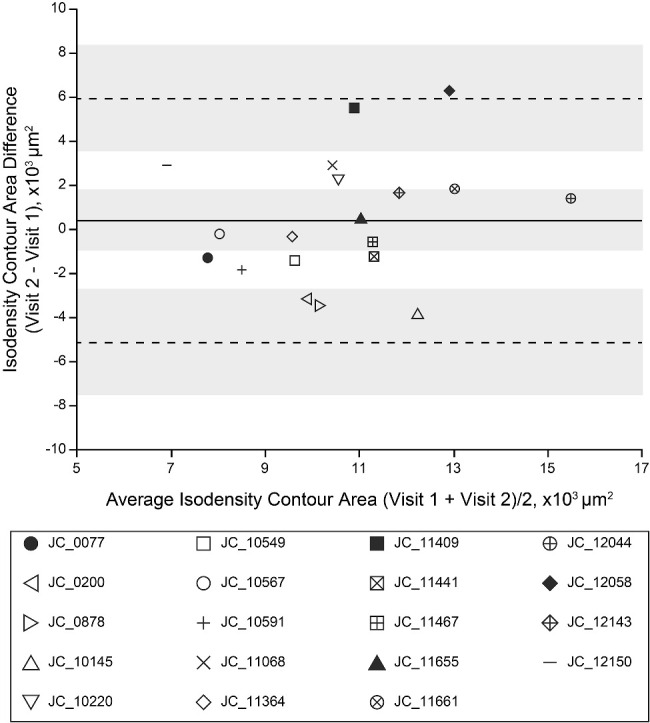
Intervisit agreement of the 80th percentile isodensity contour area. Bland–Altman analysis showed variable agreement between visit 1 and visit 2 80th percentile isodensity contour areas. The *solid line* represents the mean bias of 451 µm^2^, and the *dashed lines* represent the upper (6010 µm^2^) and lower (−5110 µm^2^) limits of agreement. *Shaded regions* represent the 95% CI about the mean bias and the limits of agreement.^38^ Each individual is indicated by a different symbol; the four *filled symbols* represent individuals included in Figure 3 and Supplementary Figure S1.

The average 80th percentile isodensity contour area was 10,400 ± 2210 µm^2^ at visit 1, and it was 10,800 ± 2750 µm^2^ at visit 2. As observed for the density values, these areas were not significantly different between visits (*t* = 0.69; *df* = 18; *P* = 0.50, paired *t*-test), although there was only a weak non-significant correlation between the two time points, in contrast to that observed for density data (Pearson correlation *r* *=* 0.36; 95% CI, −0.11 to 0.70; *P* = 0.13). Bland–Altman analysis revealed good intervisit agreement (Fig. 2), as the 95% CI of the mean difference between visits included zero (mean bias = 451 µm^2^; 95% CI, −918 to 1820 µm^2^). There was no significant correlation between the intervisit interval time and absolute difference in the 80th percentile isodensity contour area (Pearson correlation *r* *=* 0.16; 95% CI, −0.32 to 0.57; *P* = 0.52).

### Examples of Changes in Foveal Cone Topography Over Time

**Figure 3.**
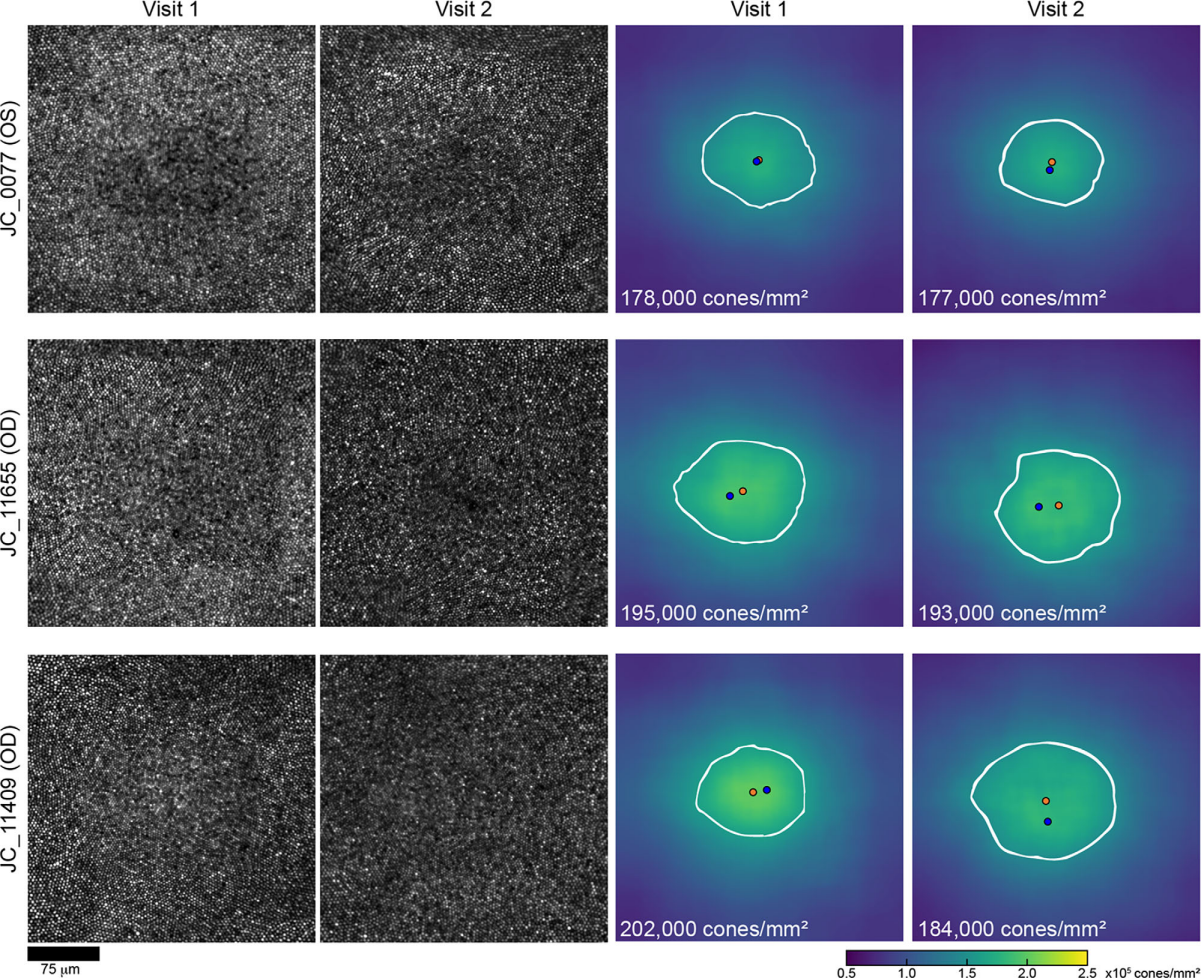
Examples of changes in foveal cone density. Shown are the foveal cone mosaics from visit 1 and visit 2 for three participants ordered from top to bottom by the least to the greatest amount of change observed in linear PCD from visit 1 to visit 2. JC_0077 showed a 0.56% change in PCD, JC_11655 showed a 1.0% change in PCD, and JC_11409 showed an 8.9% change. The average absolute change seen in all participants was 4.76% for linear PCD. The *blue dot* on the density maps represents the PCD location, the *orange dot* indicates the CDC location, and the *white outline* represents the 80th percentile isodensity contour. *Scale bar*: 75 µm.

Although there were no significant differences in foveal cone topography on average, there were examples of individuals showing relatively large changes. Shown in Figure 3 are foveal ROIs and corresponding density maps from both visits for three participants representing the range of changes observed. Notably, participant JC_12058 was found to have the largest apparent decline in foveal cone density over their 3.06-year follow-up period (linear PCD decreased by 13.6% and linear density at their CDC decreased by 16.8%; see Supplementary Fig. S1). One possibility is that changes in axial length could result in changes in cone packing,^28^^,^^39^ and this participant did have a 0.15-mm increase in axial length at visit 2 compared to their first visit. However, two participants (JC_0077 and JC_11665) had a greater increase in axial length and showed much smaller differences in their linear PCD (0.56% and 1.0%, respectively) and angular PCD (2.6% and 2.2%, respectively). A third participant (JC_0878) was the youngest participant in our cohort and a young juvenile at both visits (12 and 15 years old at visits 1 and 2, respectively). They also showed a greater increase in axial length than JC_12058 and showed a smaller difference in linear PCD over their follow-up period (9.71%), although they had a comparable difference in angular PCD (17.4%). Additionally, no significant correlation was noted between axial length differences and raw PCD differences across all participants with regard to linear density (Pearson correlation *r* *=* 0.15; 95% CI, −0.33 to 0.56; *P* = 0.54) or angular density (Pearson correlation *r* *=* 0.43; 95% CI, −0.03 to 0.74; *P* = 0.07). These observations would not support axial length differences as explaining the observed large density differences seen in a few individuals. One possibility is that some hardware change occurred between imaging sessions that affected the image scale (and thus the density estimates). Ronchi grids are imaged at each imaging session, and no such differences were noted. Additionally, examination of available peripheral images from one individual (JC_12058) showed no difference in density (see Supplementary Fig. S1). This suggests that the observed differences are due to either errors in cone identification or real changes in the foveal cone mosaic.

**Figure 4.**
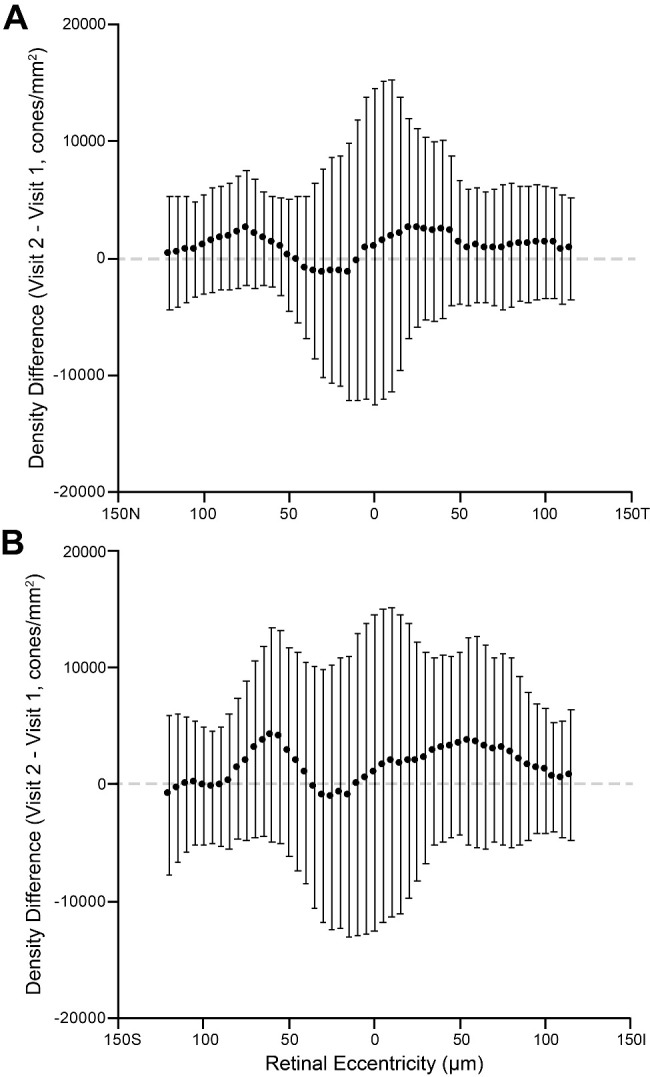
Intervisit density differences. Plotted are the average density differences along the horizontal (**A**) and vertical (**B**) meridian, extracted at the location of the CDC for each difference matrix. *Error bars* represent the SD of differences among the 19 individual difference maps at that retinal location. The *dashed line* shows zero difference for reference. No systematic differences are apparent, consistent with the comparisons of PCD and density at the CDC.

Looking at density at locations other than the singular PCD or CDC locations, we see substantial variation in the intervisit differences (Fig. 4). Along both the horizontal and vertical meridians, there was no apparent systematic difference, although it is important to note that the individual density maps were aligned to a single point (the CDC). Along both meridians, average differences were below about 5000 cones/mm^2^, or less than about 3%. The variation in density differences across subjects is lower at the more peripheral locations than the CDC center, which makes some sense, as the absolute between-subject variation in density is known to decrease away from the foveal center.

## Discussion

Due to advancements in AOSLO imaging protocols, it is now possible to visualize the central foveal cone mosaic and assess how foveal cone topography changes over time in both health and disease. As shown here, establishment of normative baseline data of these central foveal cone mosaics is vital to determine how much change in a given metric of foveal topography might be considered “normal.” Our study indicates that PCD and CDC density are fairly reproducible measurements, with about 58% of participants having less than a 5% change in PCD or CDC density between visits. This result is in accordance with minimal age-related foveal cone structural changes similarly seen in parafoveal studies by Curcio et al.,^40^ Park et al.,^41^ Jacob et al.,^42^ and Zhang et al.^43^ The stability and reproducibility of foveal cone mosaic metrics in the normal retina should aid in interpreting longitudinal changes in cone topography in patients with degenerative retinal diseases.

Despite comparable intervisit reproducibility, using the CDC to derive foveal cone density estimates may offer some advantages over the PCD. Wynne et al.^27^ showed greater reproducibility of CDC location when compared to the PCD location across different graders (the average confidence ellipse area for PCD location was 1231 µm^2^ compared to only 80 µm^2^ for CDC location). Reininger et al.^44^ also noted greater variability in intersession PCD locations (by more than threefold) when compared to CDC locations. Given this, the use of CDC may be a better anchor from which to derive retinal eccentricity in a given AOSLO montage, especially if measurements are intended to be made over time. Additionally, the CDC location may facilitate combining data from different AOSLO imaging sites, as it shows less interobserver variation in its derivation,^27^ which could help advance a multicenter database of foveal cone mosaic metrics. Consistent with previous reports,^27^^,^^44^ we observed systematically lower cone density at the CDC location than the measured PCD. Such differences would impact some studies seeking to define structure–function relationships at the human fovea.

There are some important limitations of our study. The first relates to the relatively short time between visits. With an average of just over 3 years between visits, our results are confined to this follow-up period, and extending our conclusion of stable foveal cone density metrics to longer follow-up periods would require further study. Second, we did not align the larger AOSLO montages for each participant when extracting the foveal ROIs; rather, we analyzed them independently and compared the foveal metrics (paralleling the approach that will likely be necessary in trials examining mosaics undergoing degeneration or change over time). Precise alignment of the ROIs could yield additional information regarding more subtle changes in foveal cone geometry (by assessing metrics such as nearest neighbor distance and intercell distance).

In addition, overall alignment of the montages from the two visits for each participant could provide data regarding absolute changes in the PCD and CDC locations, which was not possible in our study. A third major limitation lies in the homogeneous demographics of the participant population, which prevented the study from assessing age-related changes. Despite our ongoing recruitment efforts to increase diversity, 84% of our participant pool were less than 40 years old at visit 1 (average age, 29.89 years; range, 12–64), and they were mostly female (74%) and white (89%). Age-, sex-, and race-based differences in retinal structure have been reported using optical coherence tomography imaging,^45^^–^^47^ although this has not been systematically explored with respect to the foveal cone mosaic imaged with AOSLO. As such, our findings may not be generalizable to other populations.

Finally, we recruited individuals with clearly resolvable foveal cones from a previous study^16^ who were willing to return for additional imaging, which introduced possible selection bias. It may be that individuals with higher absolute cone density show greater changes over time due either to that topography being more susceptible to measurement error or to more frequent real changes. However, we do not view this as a major limitation, as the average PCDs in our cohort (187,000 cones/mm^2^ at visit 1 and 189,000 cones/mm^2^ at visit 2) were very similar to those reported in previous AOSLO-based studies.^18^^,^^20^^,^^23^^,^^28^^,^^29^ Also, as noted earlier, the metrics chosen are commonly used in the field, with density being perhaps the most commonly reported metric in studies describing cone mosaic topography.^15^ That said, it is worth highlighting that density is known to have average sensitivity in detecting changes in cone numerosity.^48^ Spacing metrics such as nearest neighbor distance are more robust and may show better reproducibility, whereas regularity metrics (e.g., number of nearest neighbors regularity index) are more sensitive and would likely show worse reproducibility. The anticipated change in the cone mosaic along with the sensitivity of a given metric should be used together when choosing how to monitor the cone mosaic over time in future clinical trials.

## Conclusions

In conclusion, our study provides important reproducibility data regarding foveal cone topography in participants with normal vision. Larger studies in more diverse populations are necessary and may help determine the extent of foveal changes that may arise due to normal aging processes, as well as those that may occur during postnatal aspects of foveal development. These studies may also uncover the cause of the observed differences in cone density seen in some individuals. Additionally, our data could be used as a benchmark to interpret longitudinal findings in patients with inherited retinal degenerations.

## Supplementary Material

Supplement 1

Supplement 2

Supplement 3
