# Supplementary material for: Intervisit Reproducibility of Foveal Cone Density Metrics
Source: Transl Vis Sci Technol. 2024 Jun 24;13(6):18. doi: 10.1167/tvst.13.6.18 (PMC11205225; doi:10.1167/tvst.13.6.18)
Supplement: Supplement 1 [file tvst-13-6-18_s001.pdf]

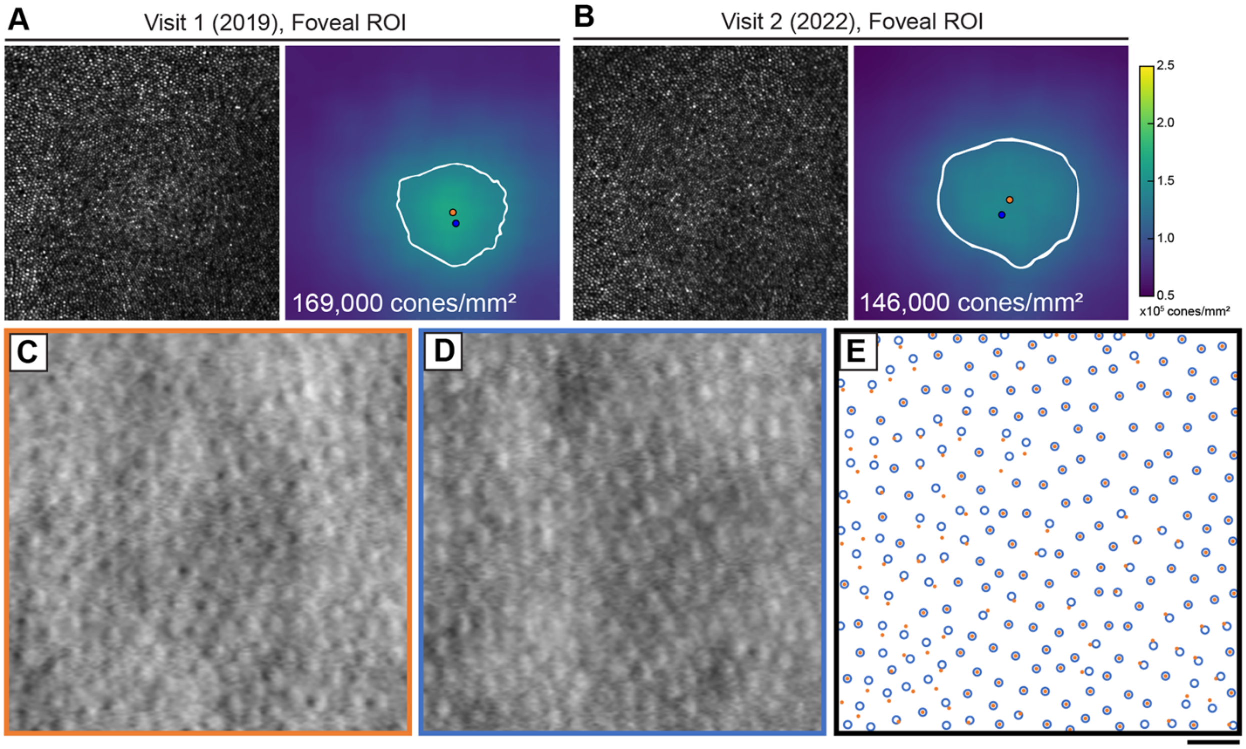

Supplementary Figure S1. Assessing cone mosaic changes in JC_12058. (**A**) Foveal ROI and corresponding density map from visit 1, with a PCD of 169,000 cones/mm^2. The blue dot on the density maps represents the PCD location, the orange dot indicates the CDC location, and the white outline represents the 80th percentile isodensity contour. (**B**) Foveal ROI and corresponding density map from visit 1, with a PCD of 146,000 cones/mm^2. Density map markers are the same as in A. We were interested to see if density changes were restricted to the fovea or if they were seen across the macula (as macula-wide differences might be explained by a methodological error).{Heitkotter, 2021 #12598} We manually aligned the entire AOSLO montage from each visit and then extracted a 0.35 x 0.35º ROI at 9º superior to the fovea from each montage (visit 1, **C** and visit 2, **D**). The use of split-detection enables reliable identification of cone inner segments, whereas the confocal image makes it difficult to disambiguate rod from cone structure at these retinal locations. The bound cone densities were 10,500 cones/mm^2 (981 cones/deg^2) at visit 1 and 10,800 cones/mm^2 (1,020 cones/deg^2) at visit 2. The change in linear PCD (3.9% increase) is much lower and in the opposite direction than that observed at the fovea (14.7% decrease), consistent with mosaic changes being restricted to their fovea. Further evidence of minimal change in this parafoveal region comes from the near-perfect alignment of individual cones (**E,** Orange dots are cone markings from visit 1 and the blue empty circles are cone markings from visit 2). Accordingly, the bound nearest neighbor distance differed by only 1.7% (visit 1: 8.31 µm, visit 2: 8.17 µm) and the intercell distance differed by only 1.5% (visit 1: 10.8 µm, visit 2: 10.6 µm). Together these data suggest the decrease in foveal cone density in this participant is real. Scale bar is 25 microns and applies to panels **C-E**.
