# Supplementary figures and images for: Intervisit Reproducibility of Foveal Cone Density Metrics
Source: Transl Vis Sci Technol. 2024 Jun 24;13(6):18. doi: 10.1167/tvst.13.6.18 (PMC11205225; doi:10.1167/tvst.13.6.18)

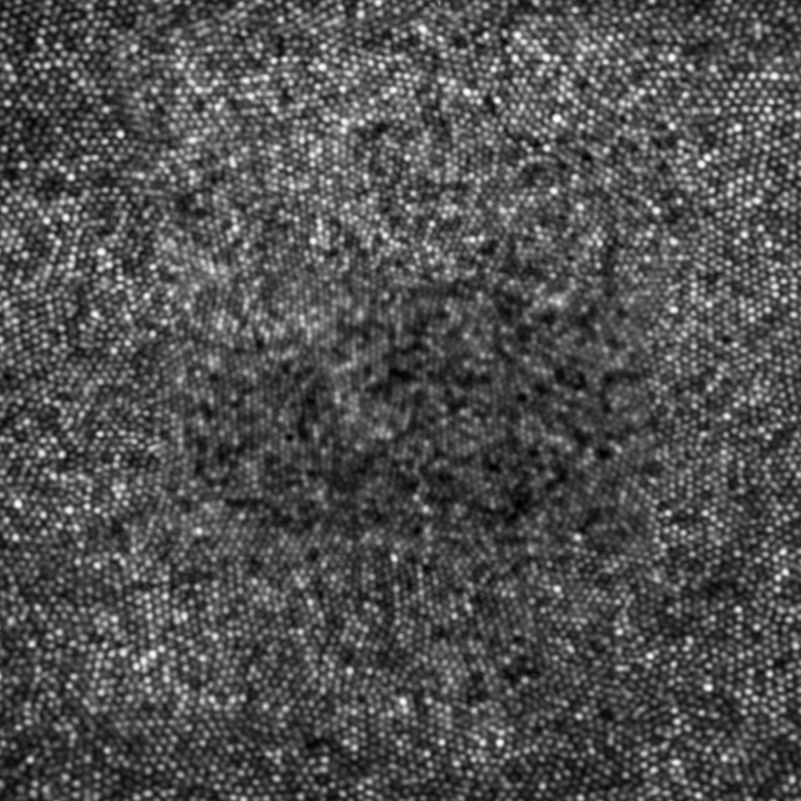

Supplement: Supplement 3 [file tvst-13-6-18_s003.zip › JC_0077_visit1_300um.tif]

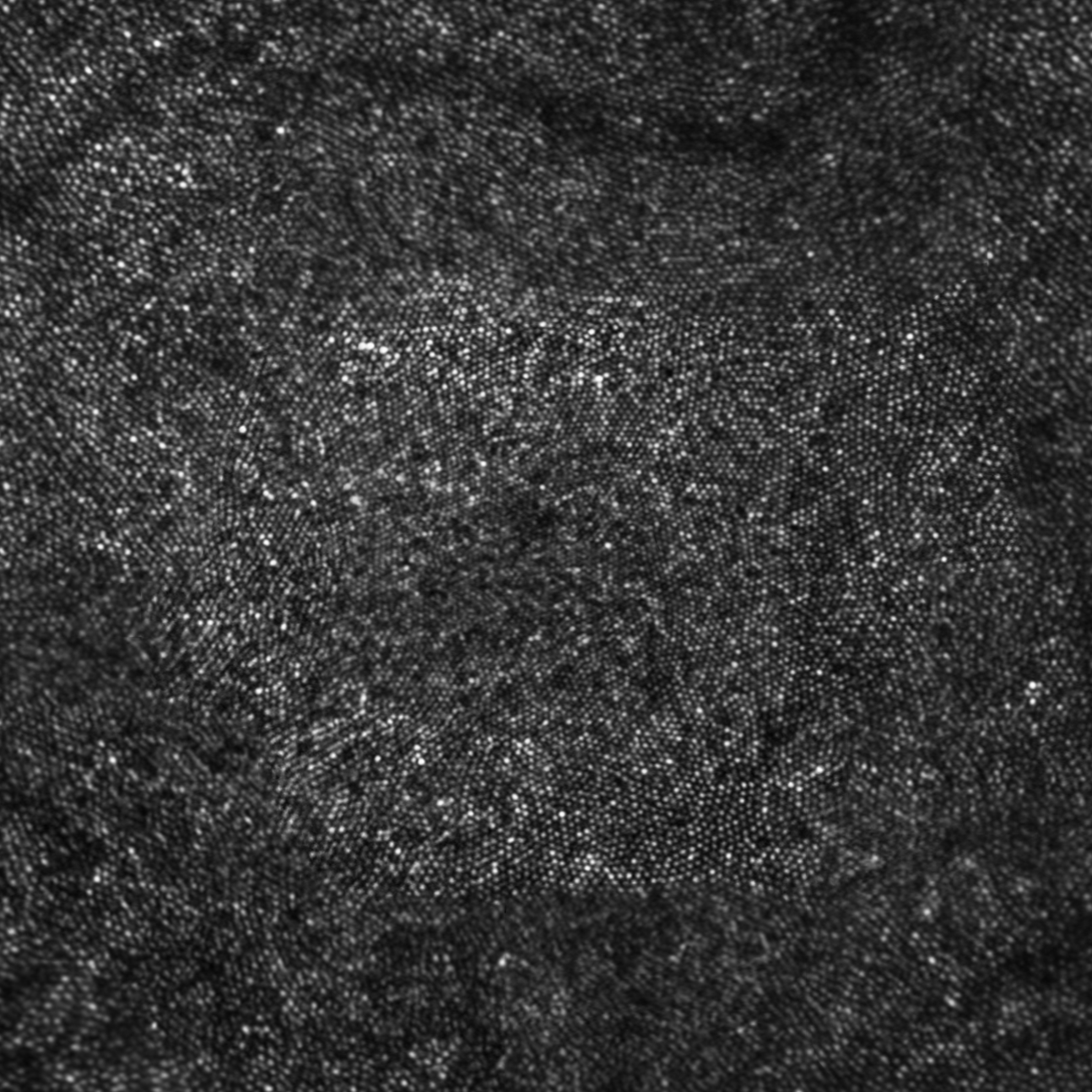

Supplement: Supplement 3 [file tvst-13-6-18_s003.zip › JC_0077_visit2_500um.tif]

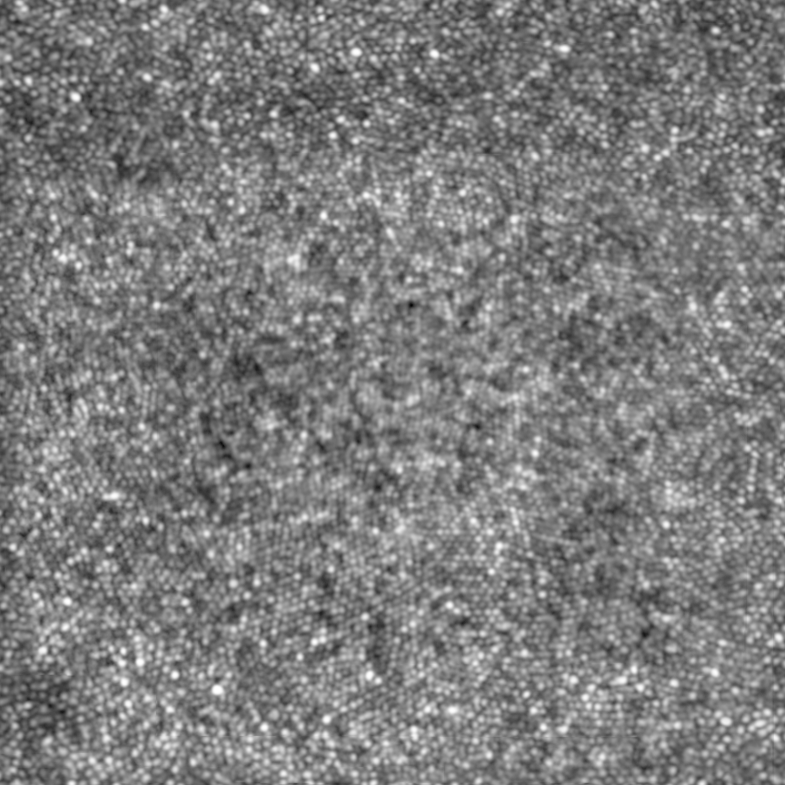

Supplement: Supplement 3 [file tvst-13-6-18_s003.zip › JC_0200_visit1_300um.tif]

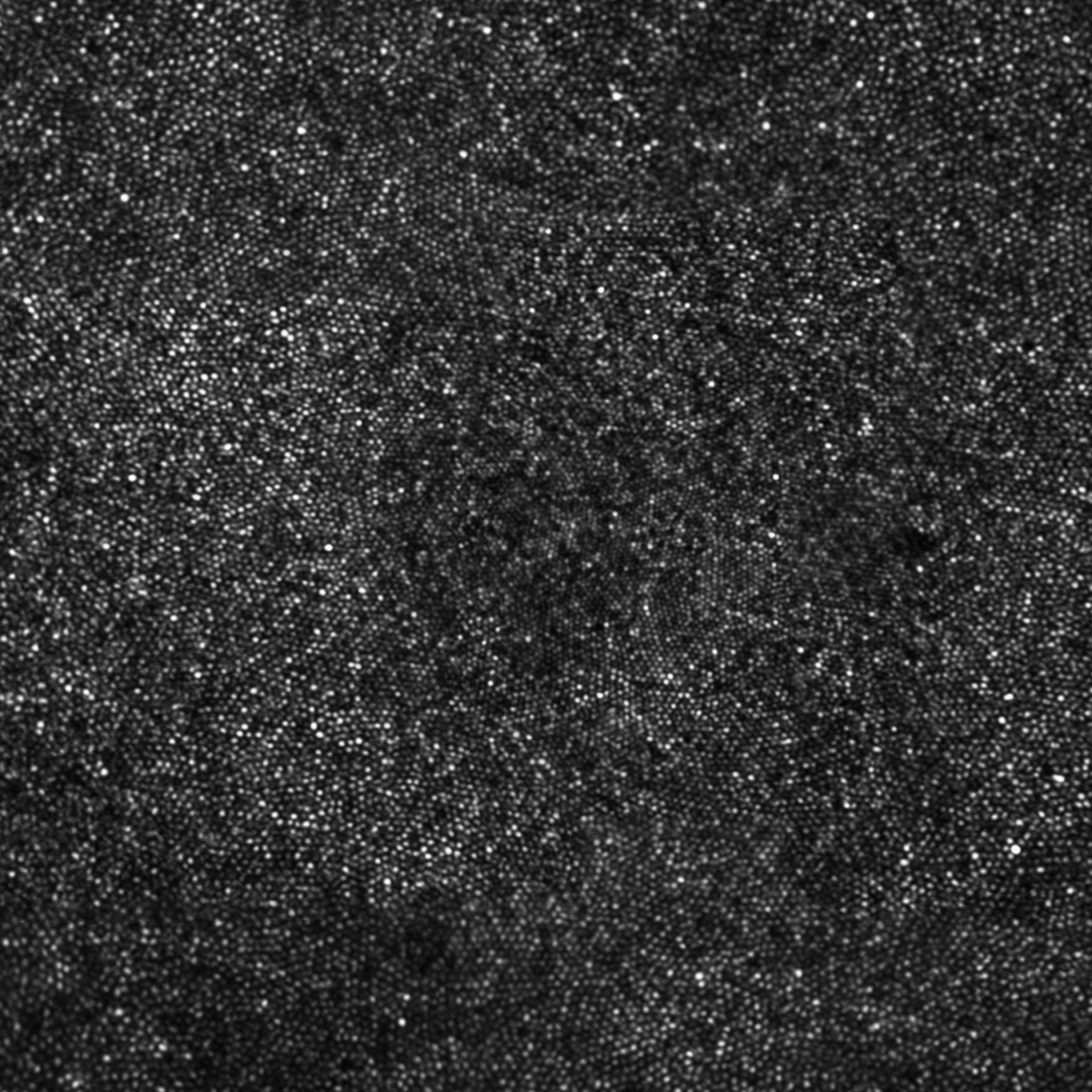

Supplement: Supplement 3 [file tvst-13-6-18_s003.zip › JC_0200_visit2_500um.tif]

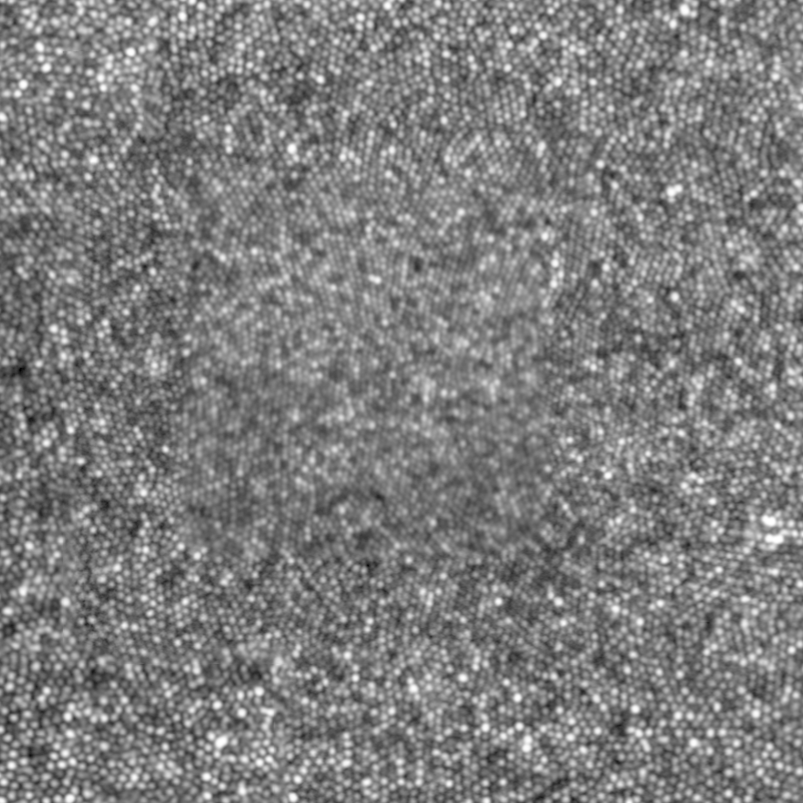

Supplement: Supplement 3 [file tvst-13-6-18_s003.zip › JC_0878_visit1_300um.tif]

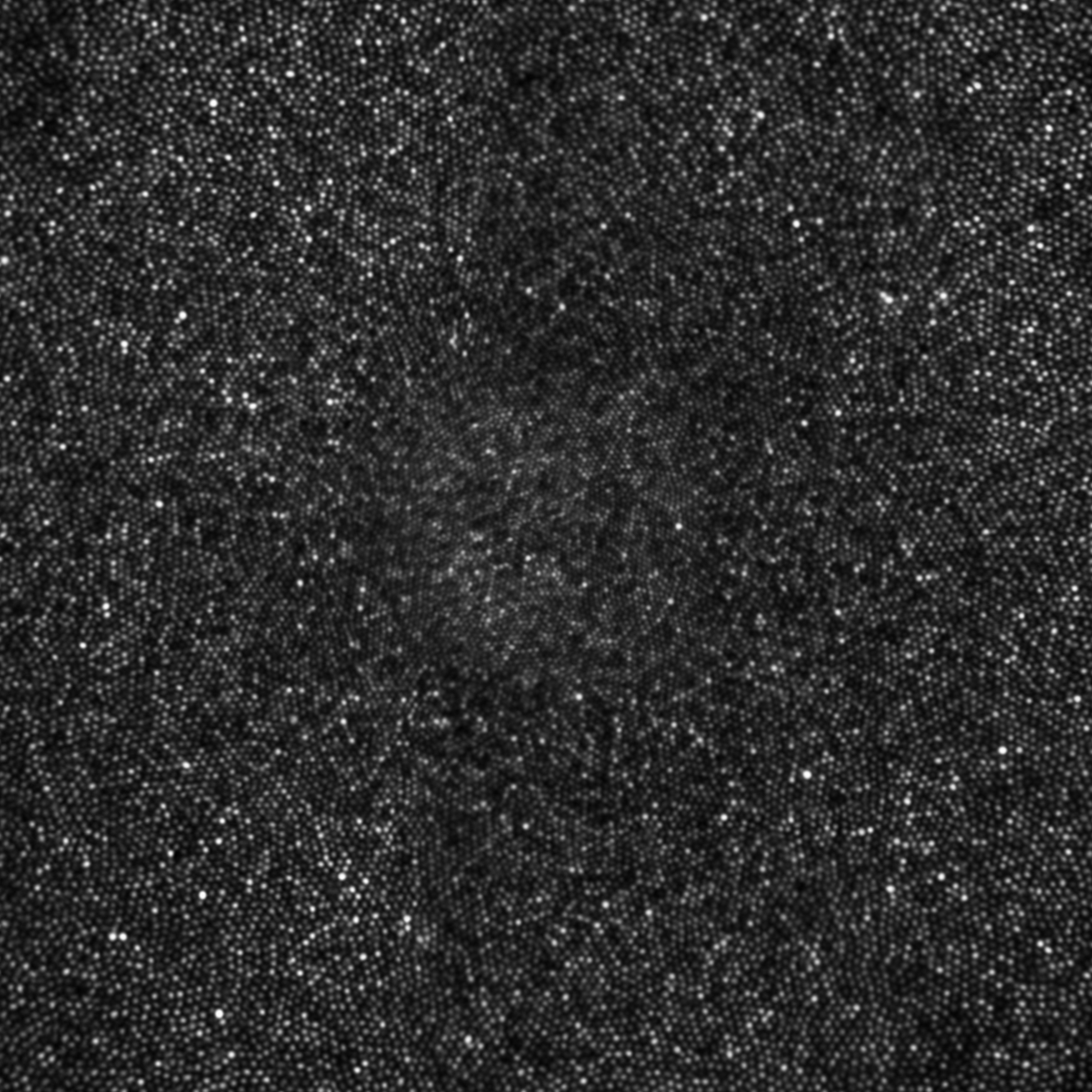

Supplement: Supplement 3 [file tvst-13-6-18_s003.zip › JC_0878_visit2_500um.tif]

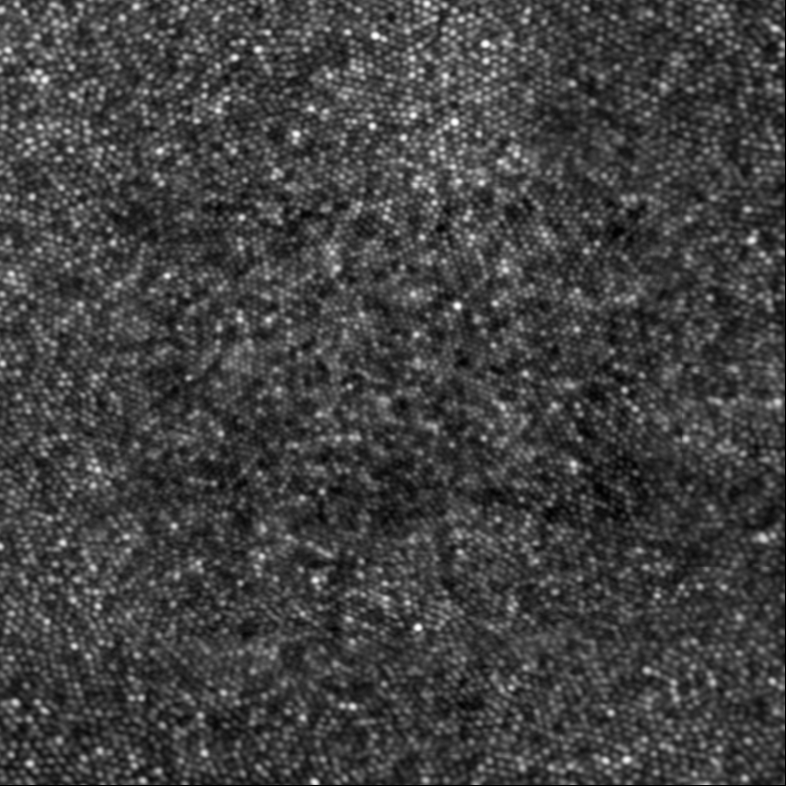

Supplement: Supplement 3 [file tvst-13-6-18_s003.zip › JC_10145_visit1_300um.tif]

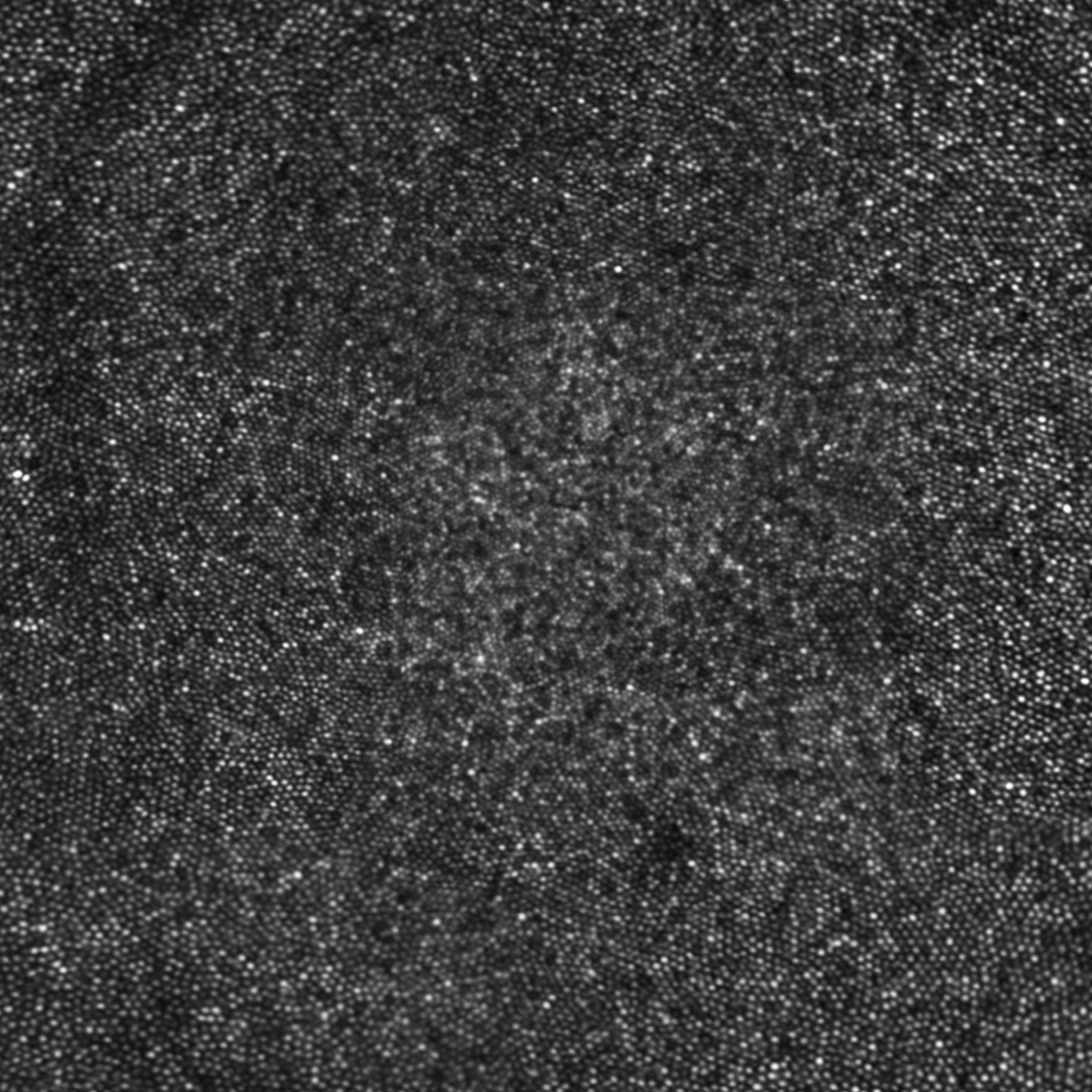

Supplement: Supplement 3 [file tvst-13-6-18_s003.zip › JC_10145_visit2_500um.tif]

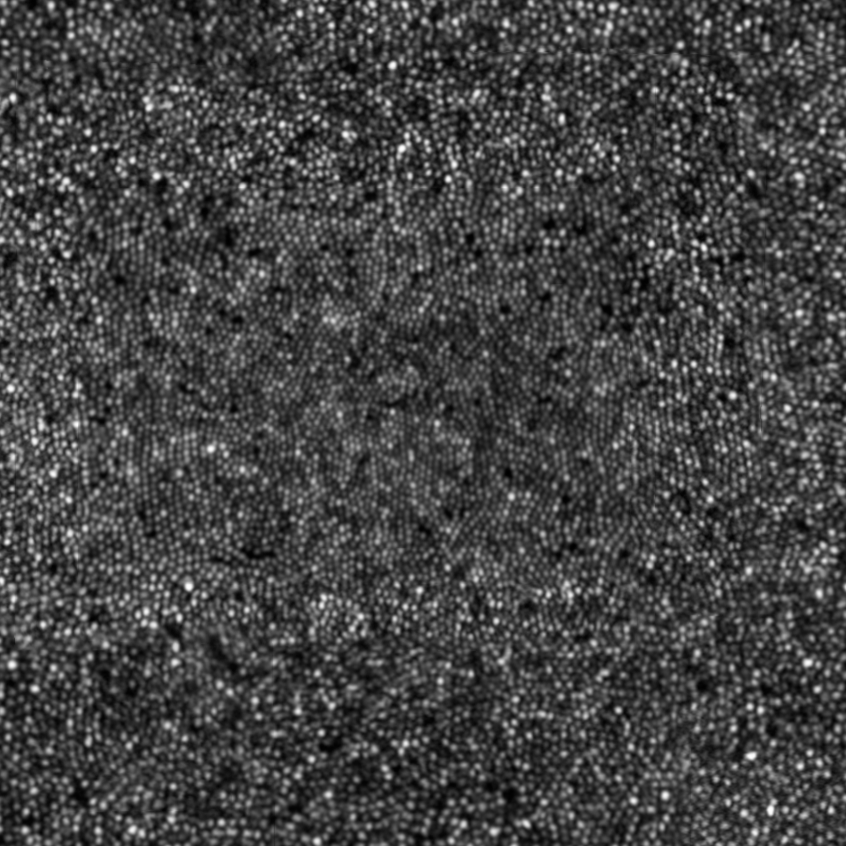

Supplement: Supplement 3 [file tvst-13-6-18_s003.zip › JC_10220_visit1_300um.tif]

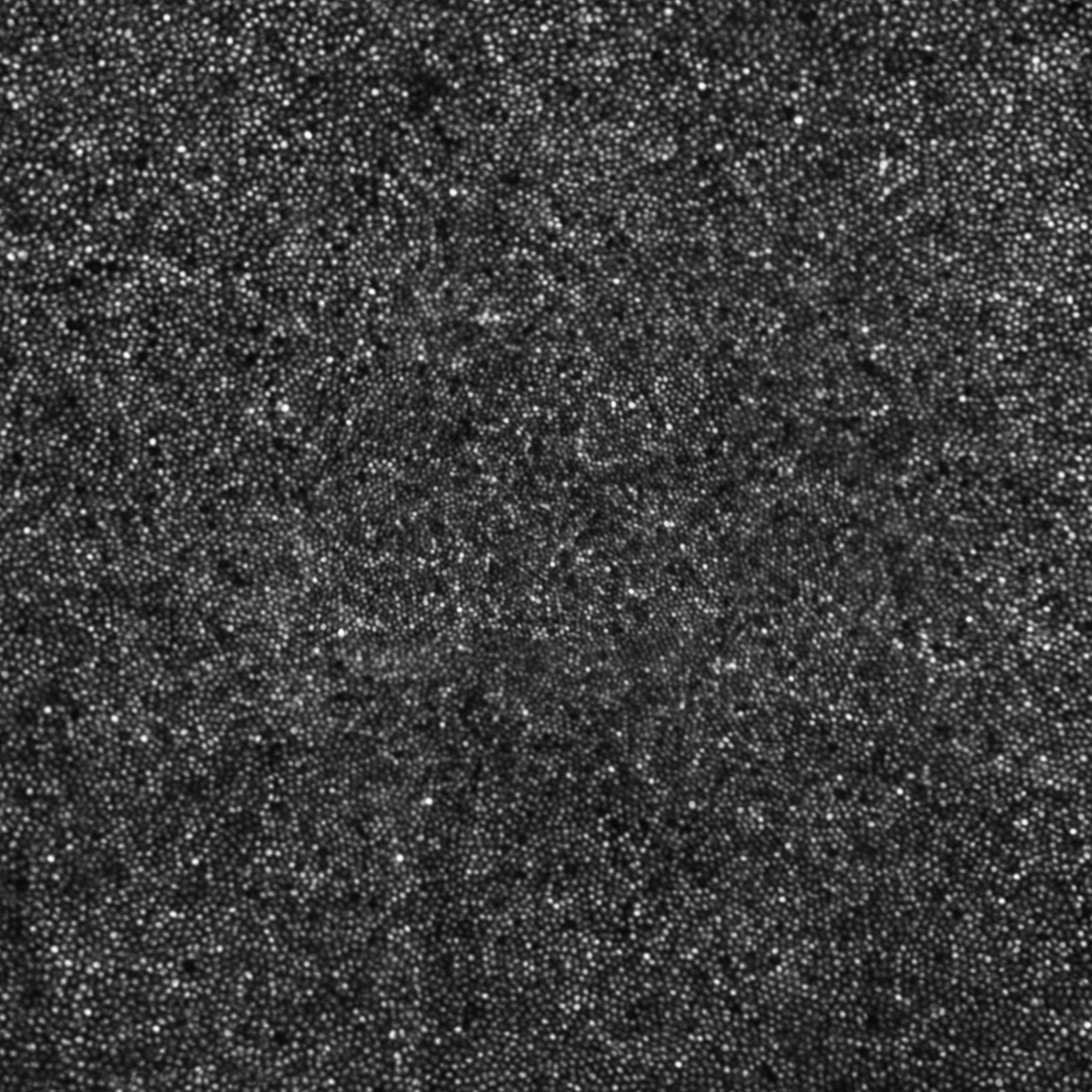

Supplement: Supplement 3 [file tvst-13-6-18_s003.zip › JC_10220_visit2_500um.tif]

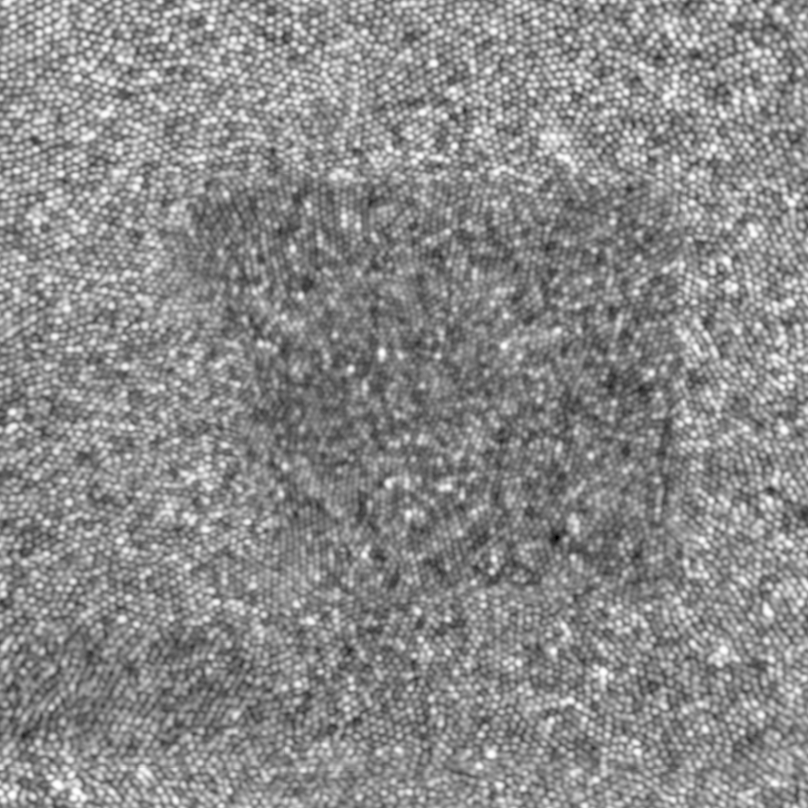

Supplement: Supplement 3 [file tvst-13-6-18_s003.zip › JC_10549_visit1_300um.tif]

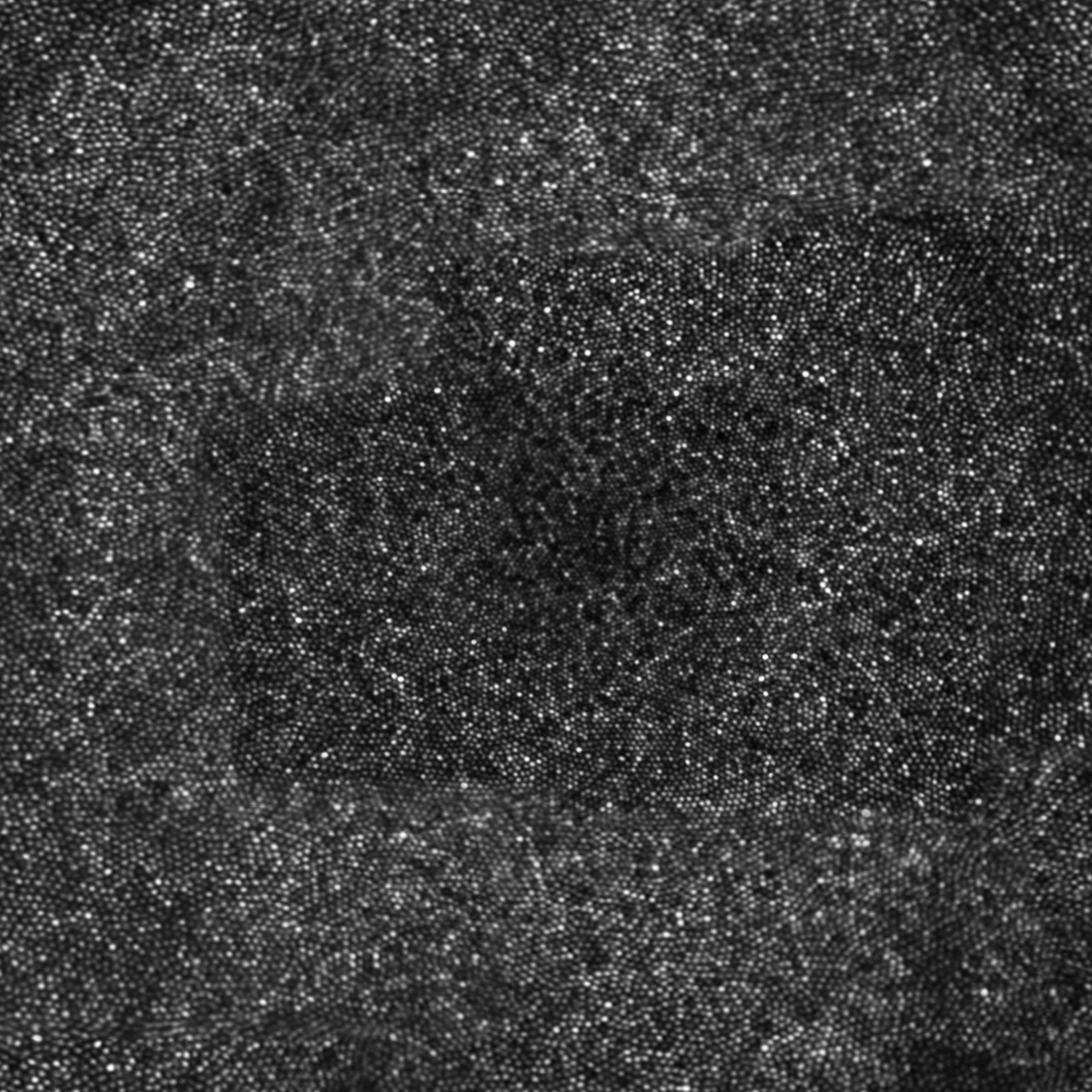

Supplement: Supplement 3 [file tvst-13-6-18_s003.zip › JC_10549_visit2_500um.tif]

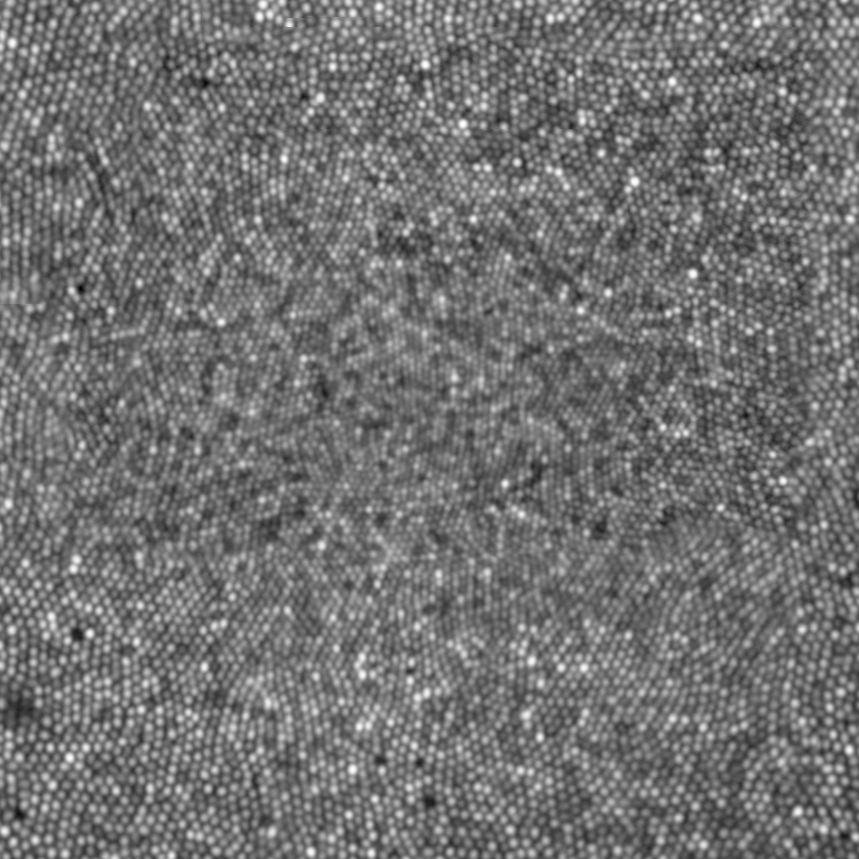

Supplement: Supplement 3 [file tvst-13-6-18_s003.zip › JC_10567_visit1_300um.tif]

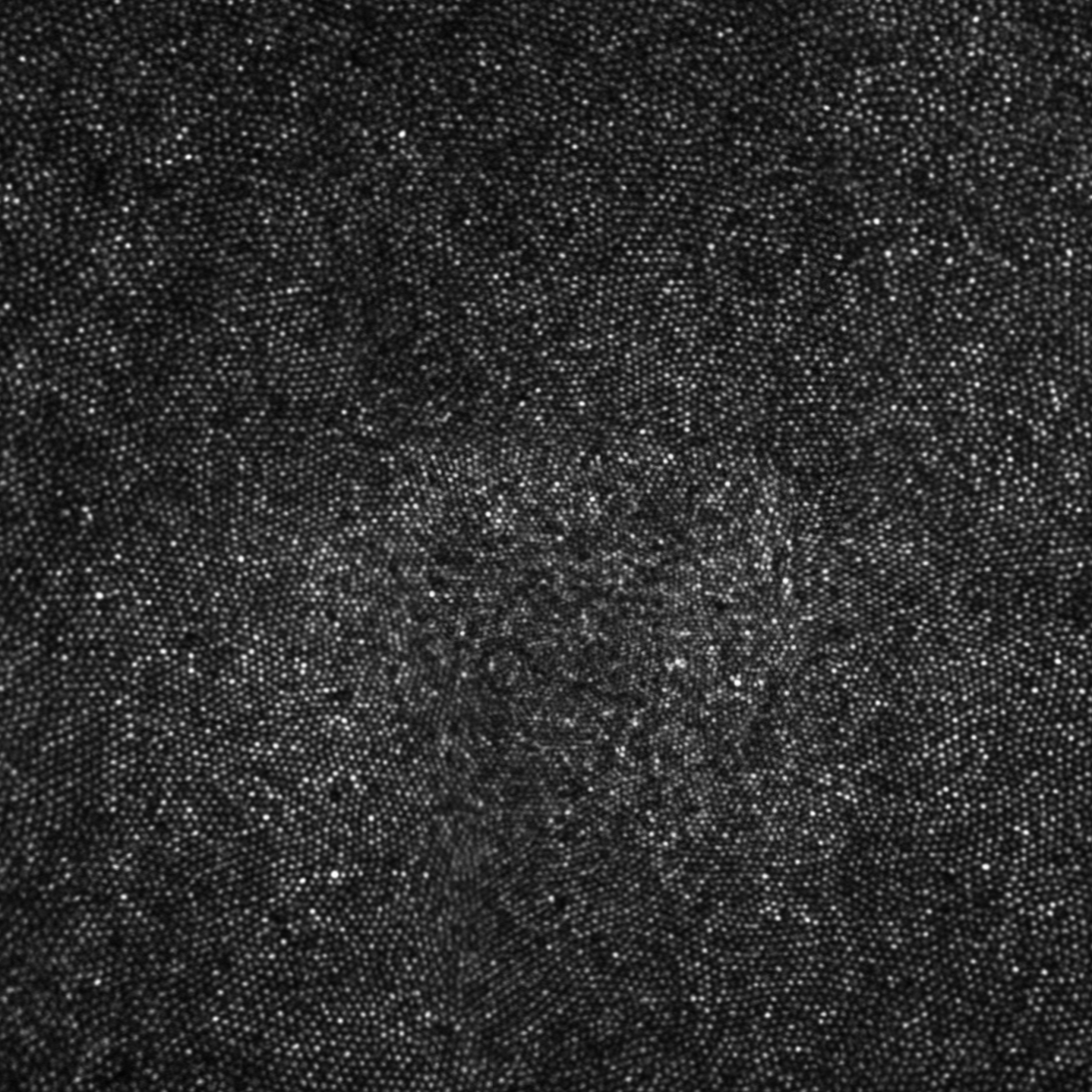

Supplement: Supplement 3 [file tvst-13-6-18_s003.zip › JC_10567_visit2_500um.tif]

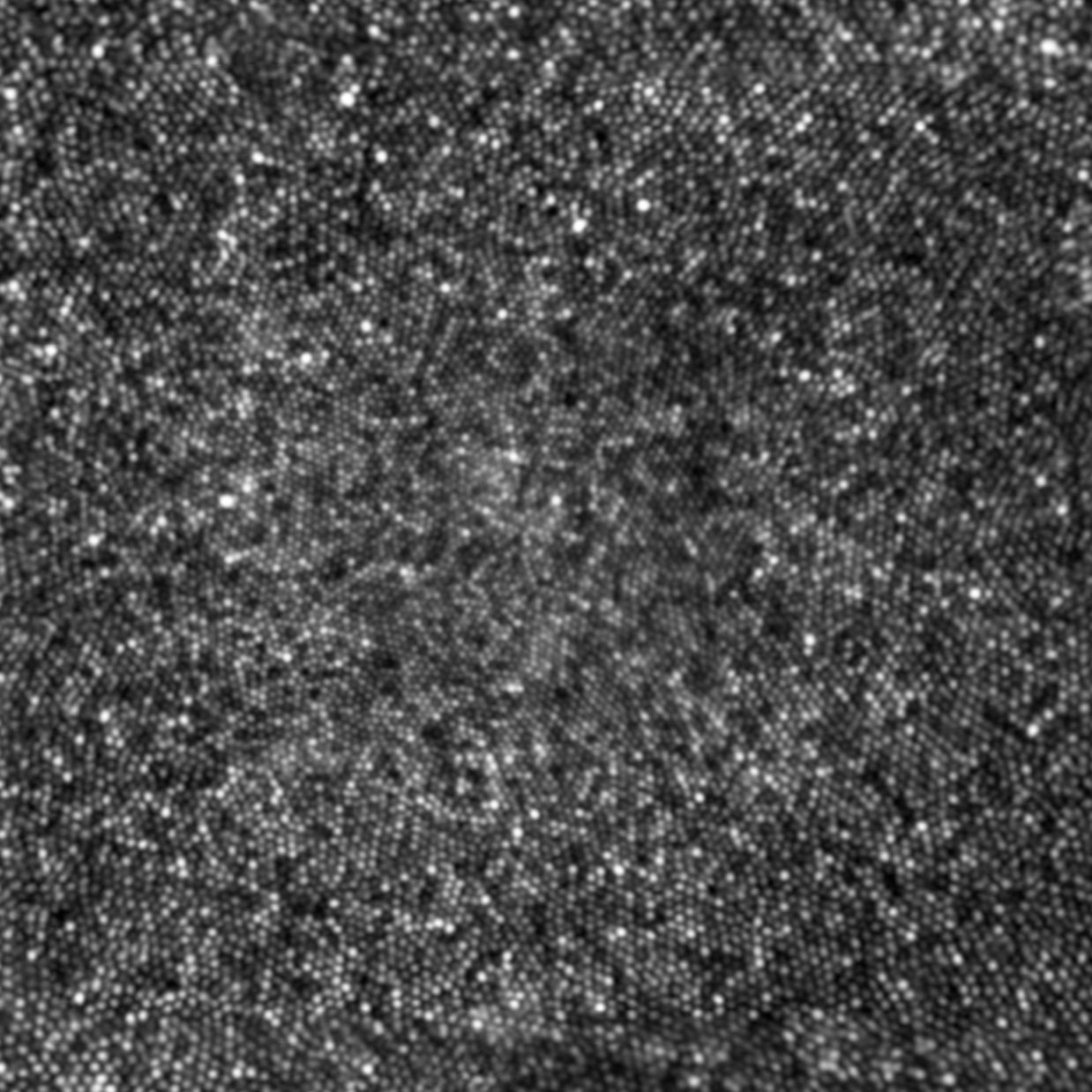

Supplement: Supplement 3 [file tvst-13-6-18_s003.zip › JC_10591_visit1_300um.tif]

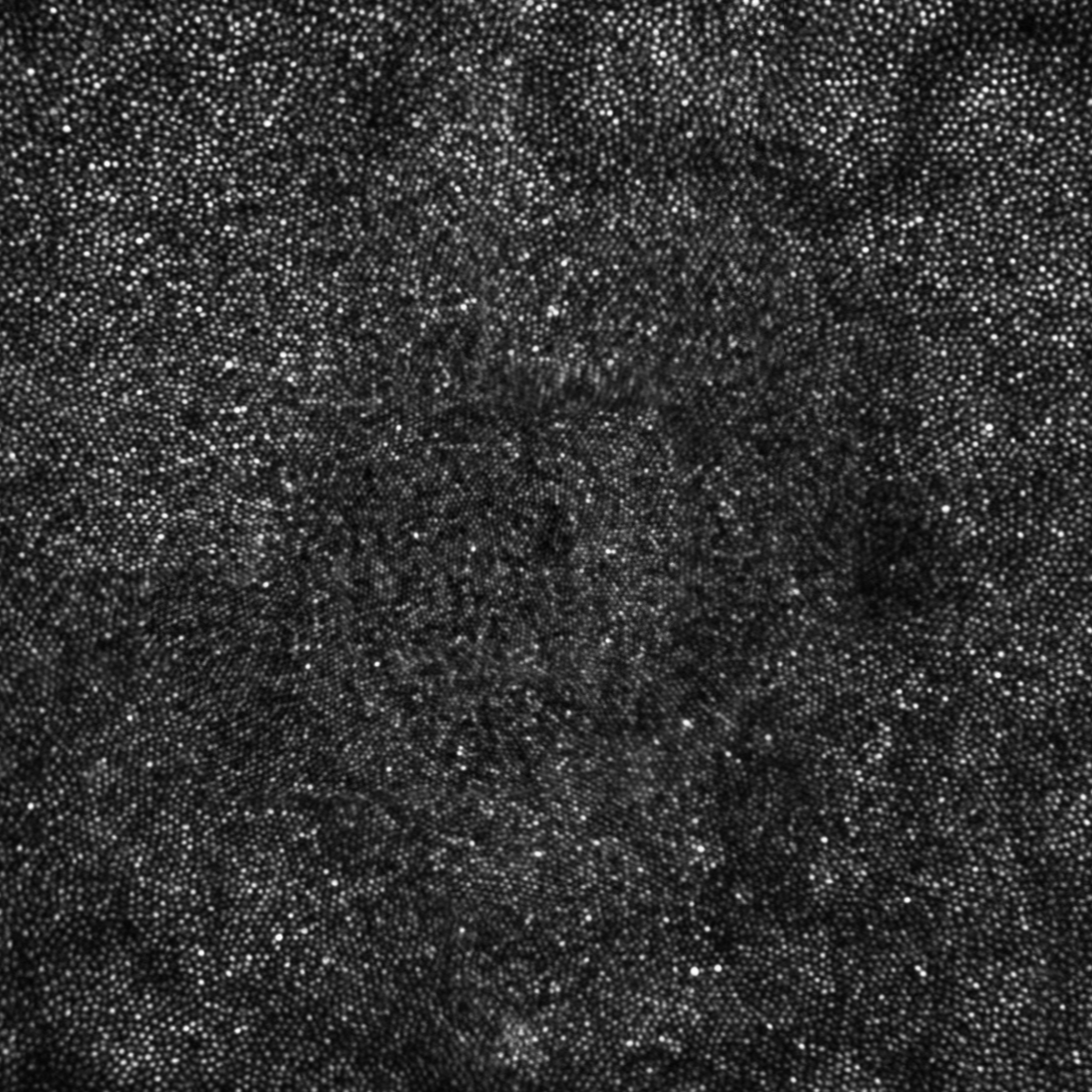

Supplement: Supplement 3 [file tvst-13-6-18_s003.zip › JC_10591_visit2_500um.tif]

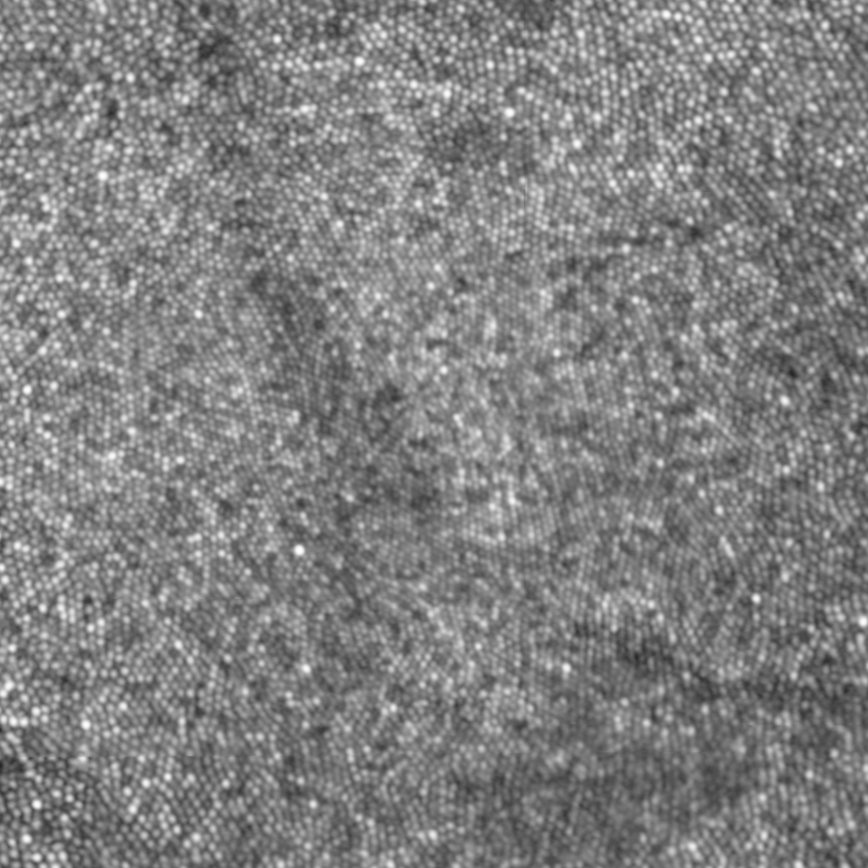

Supplement: Supplement 3 [file tvst-13-6-18_s003.zip › JC_11068_visit1_300um.tif]

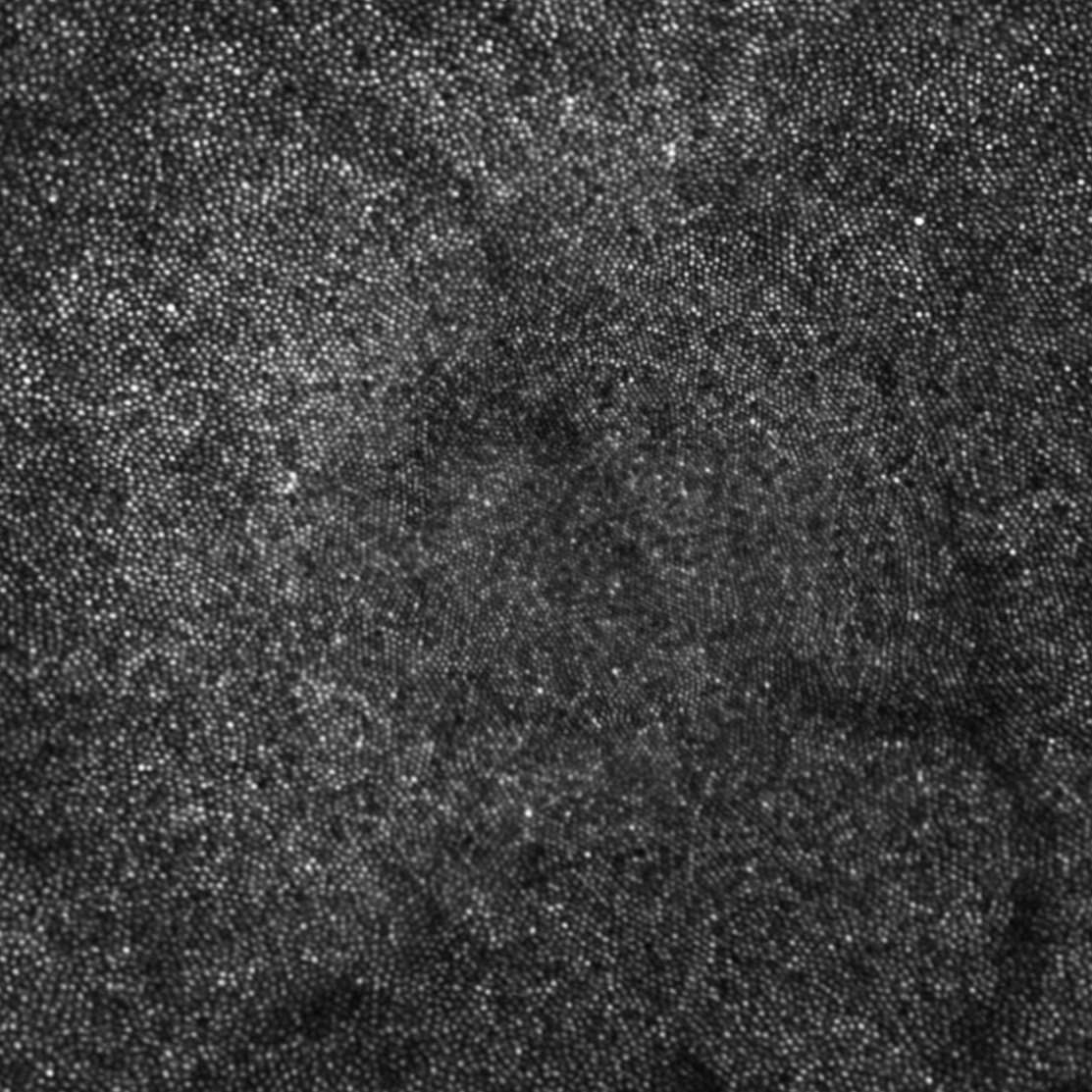

Supplement: Supplement 3 [file tvst-13-6-18_s003.zip › JC_11068_visit2_500um.tif]

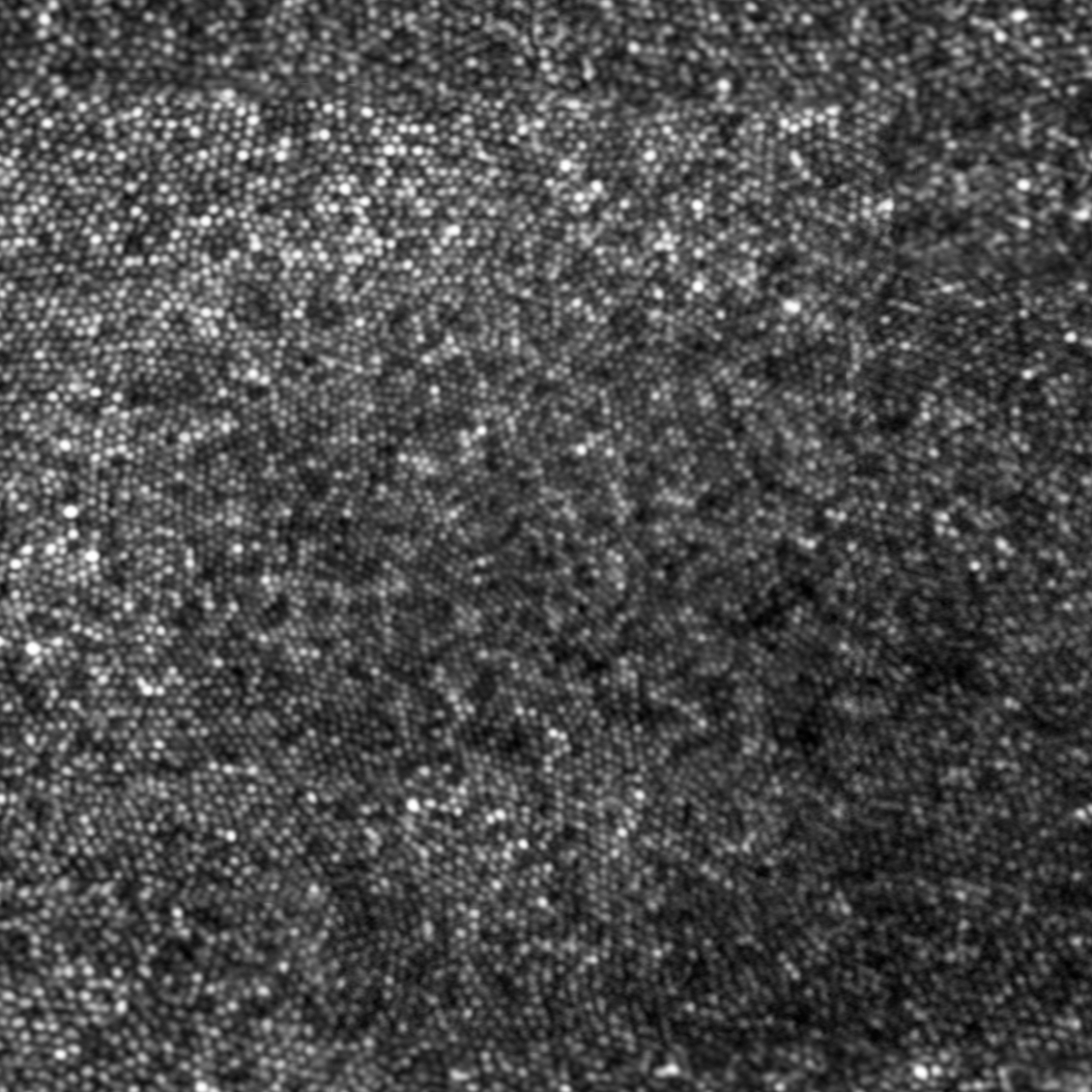

Supplement: Supplement 3 [file tvst-13-6-18_s003.zip › JC_11364_visit1_300um.tif]

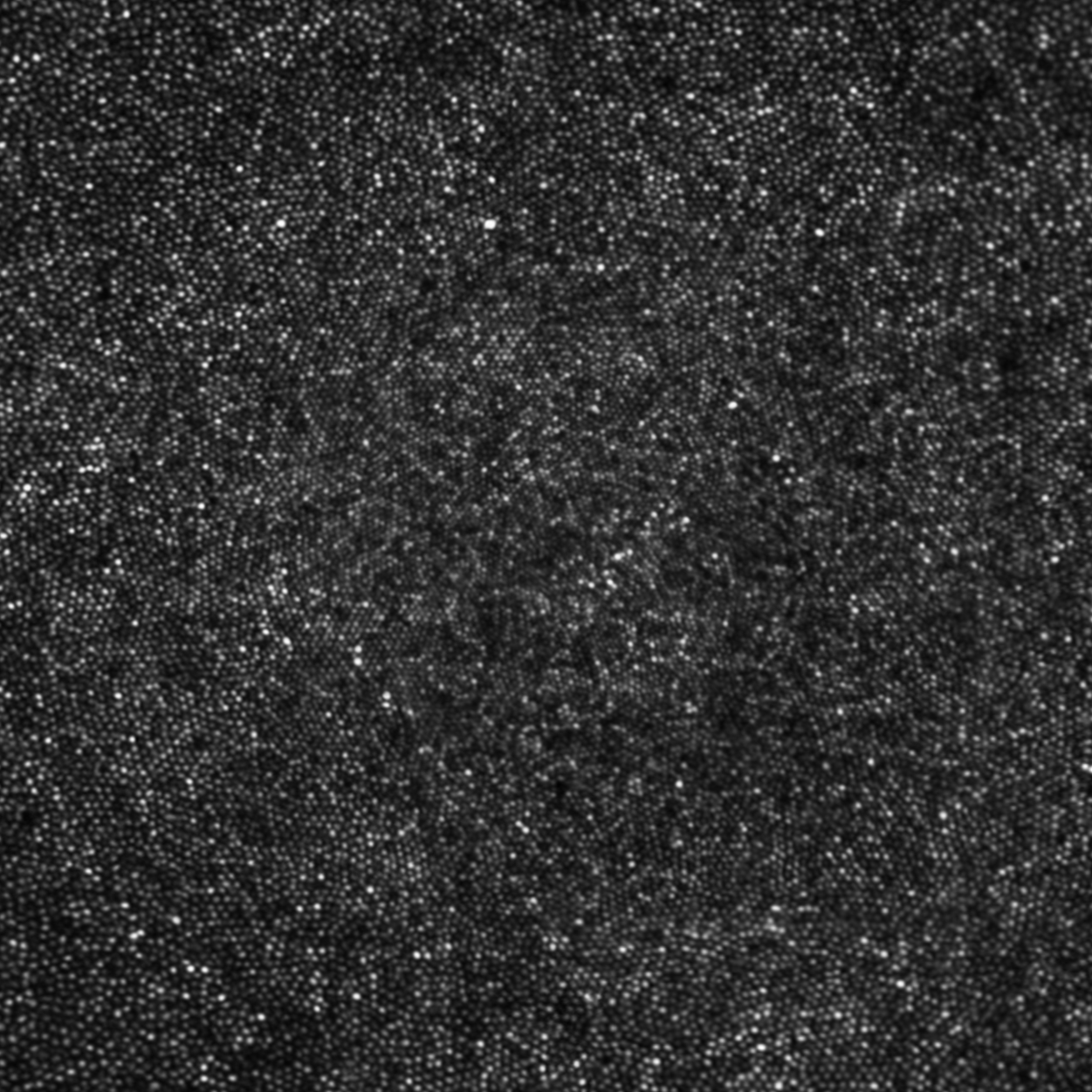

Supplement: Supplement 3 [file tvst-13-6-18_s003.zip › JC_11364_visit2_500um.tif]

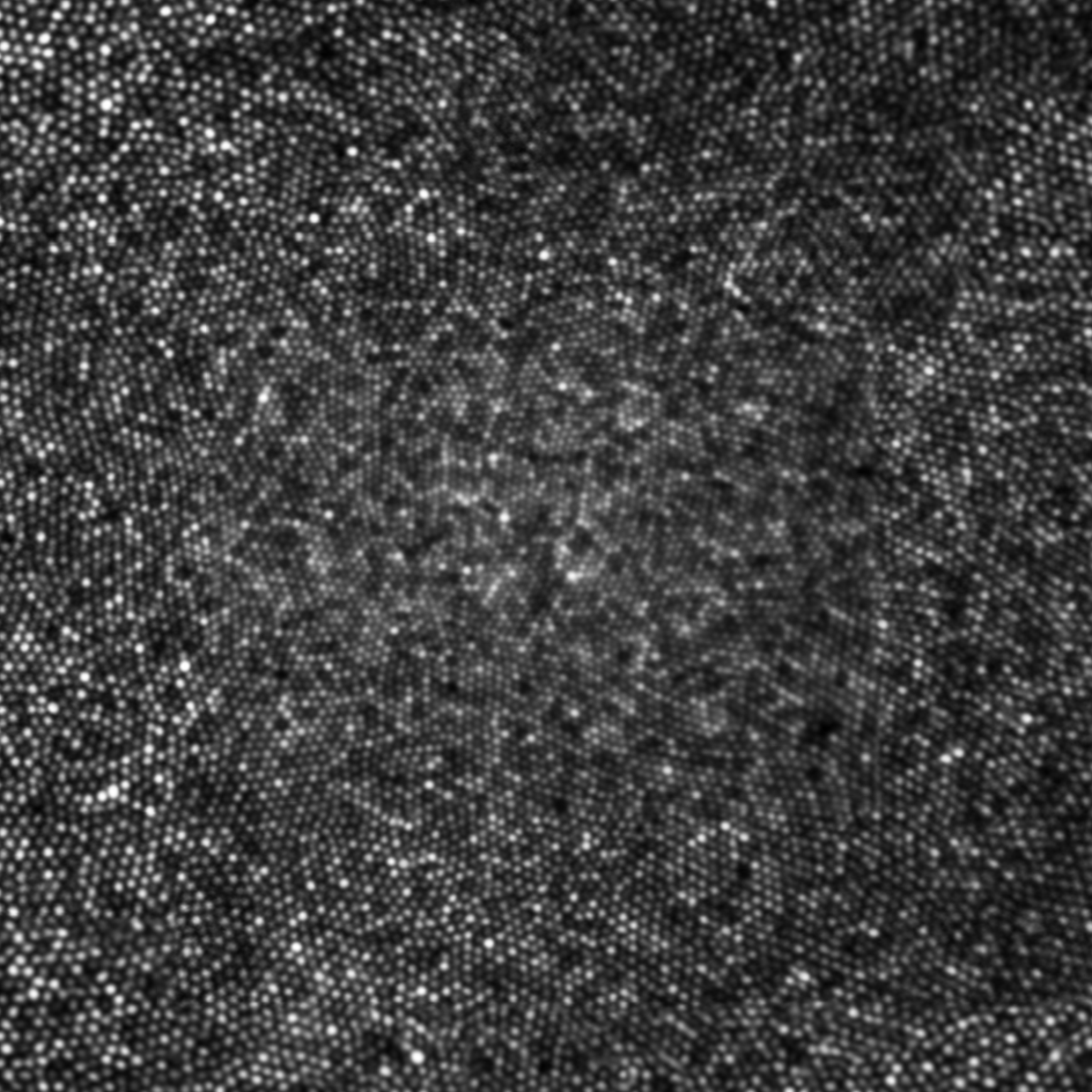

Supplement: Supplement 3 [file tvst-13-6-18_s003.zip › JC_11409_visit1_300um.tif]

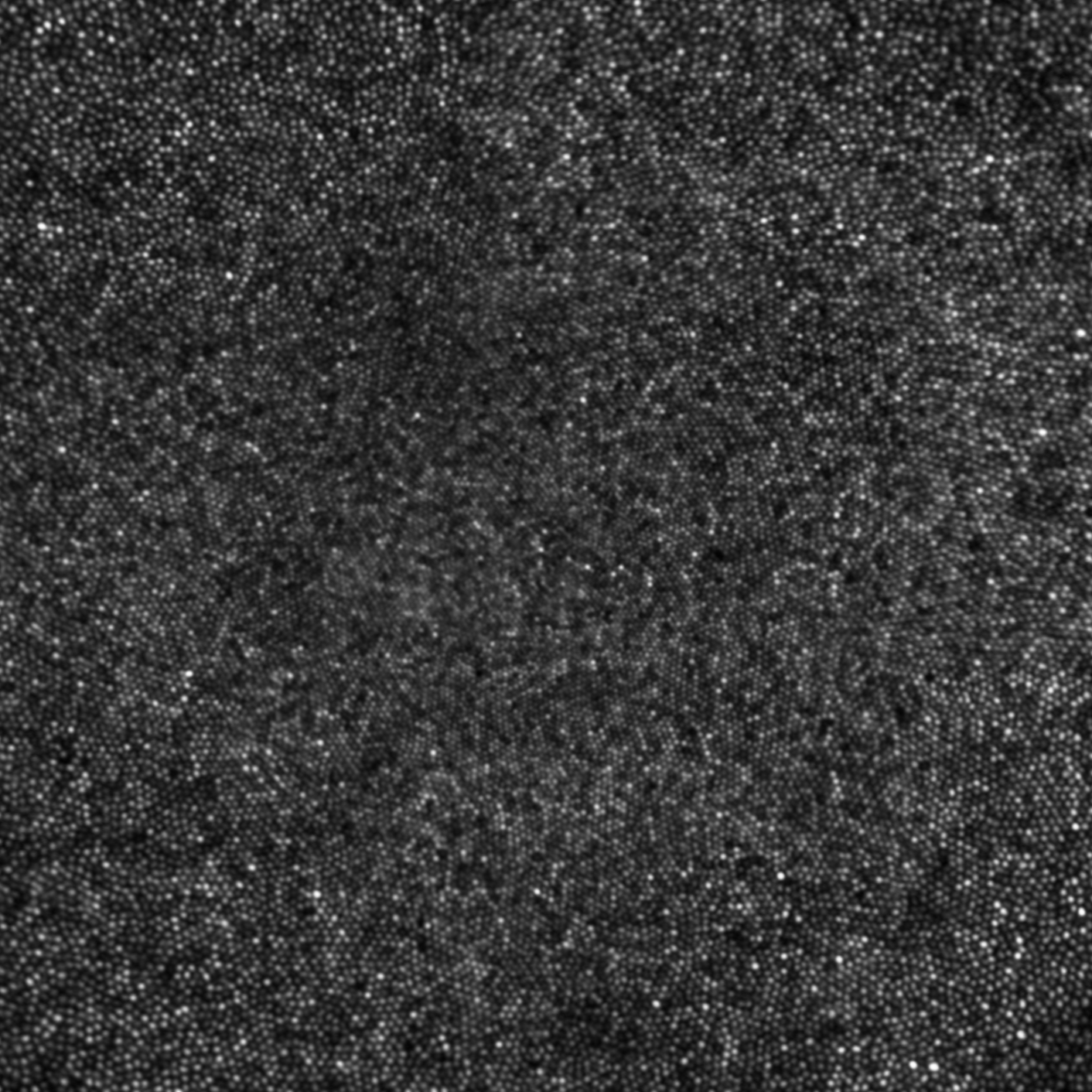

Supplement: Supplement 3 [file tvst-13-6-18_s003.zip › JC_11409_visit2_500um.tif]

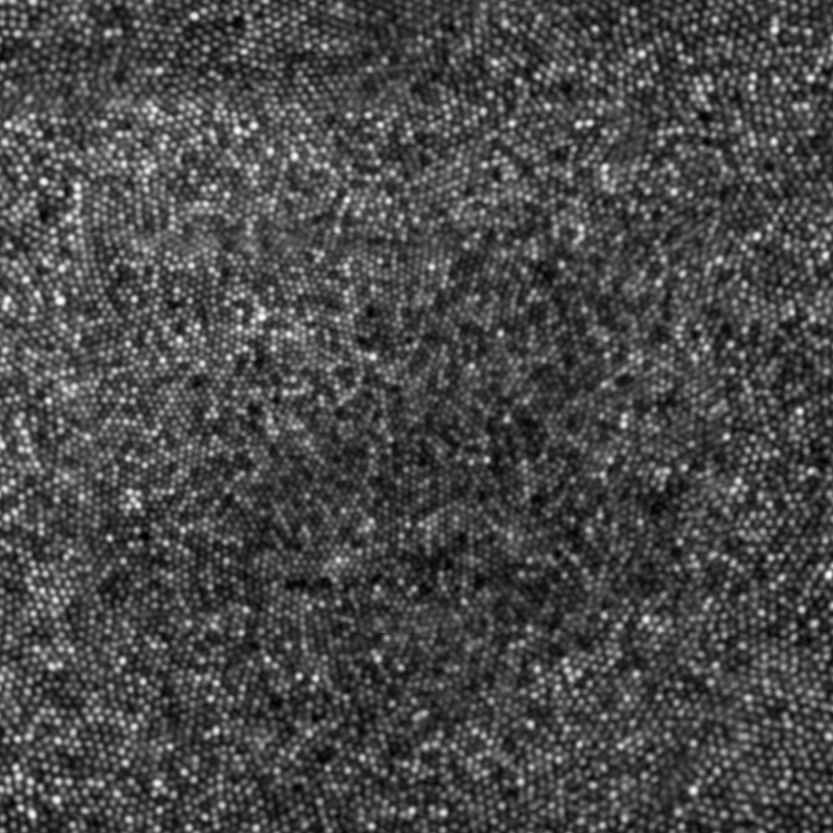

Supplement: Supplement 3 [file tvst-13-6-18_s003.zip › JC_11441_visit1_300um.tif]

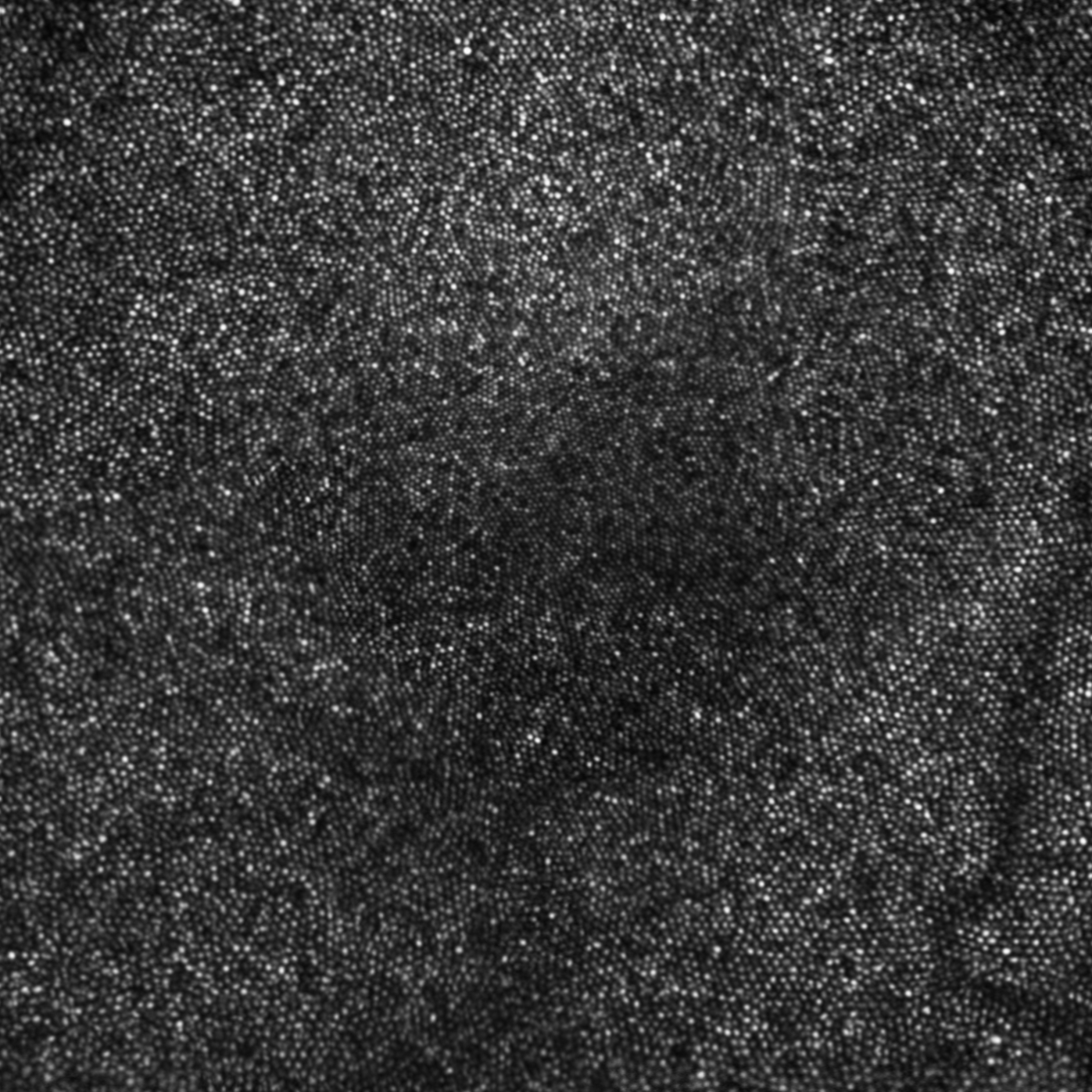

Supplement: Supplement 3 [file tvst-13-6-18_s003.zip › JC_11441_visit2_500um.tif]

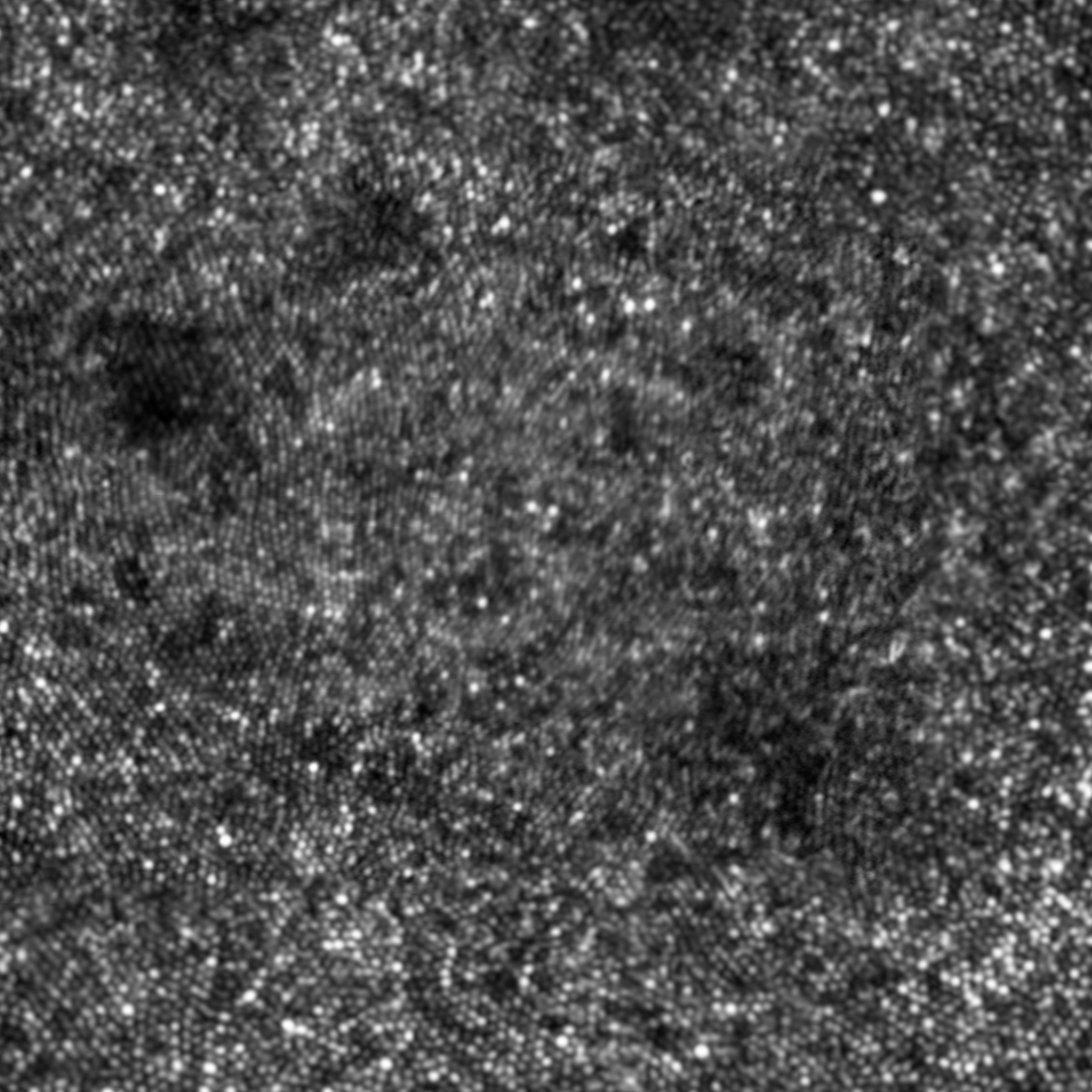

Supplement: Supplement 3 [file tvst-13-6-18_s003.zip › JC_11467_visit1_300um.tif]

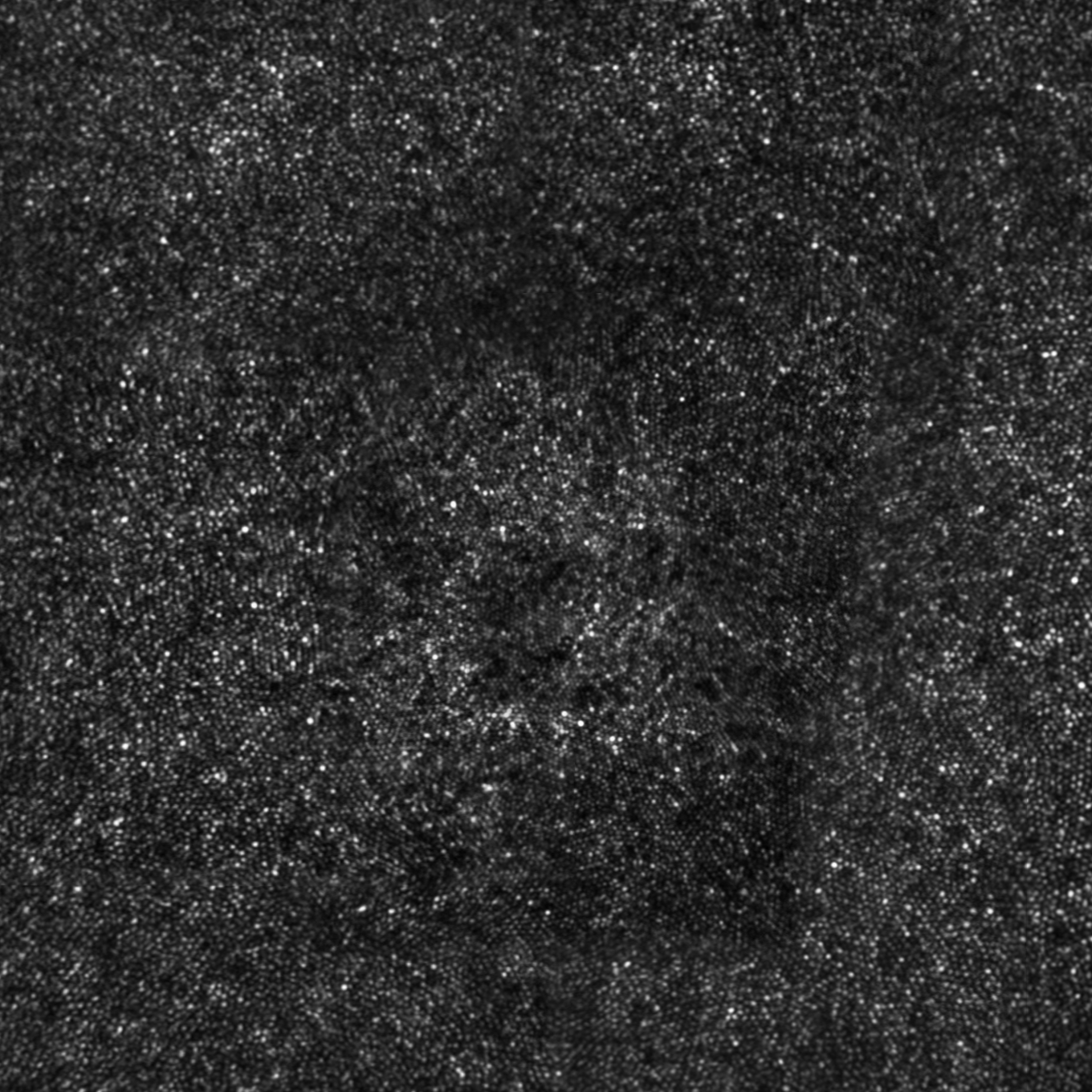

Supplement: Supplement 3 [file tvst-13-6-18_s003.zip › JC_11467_visit2_500um.tif]

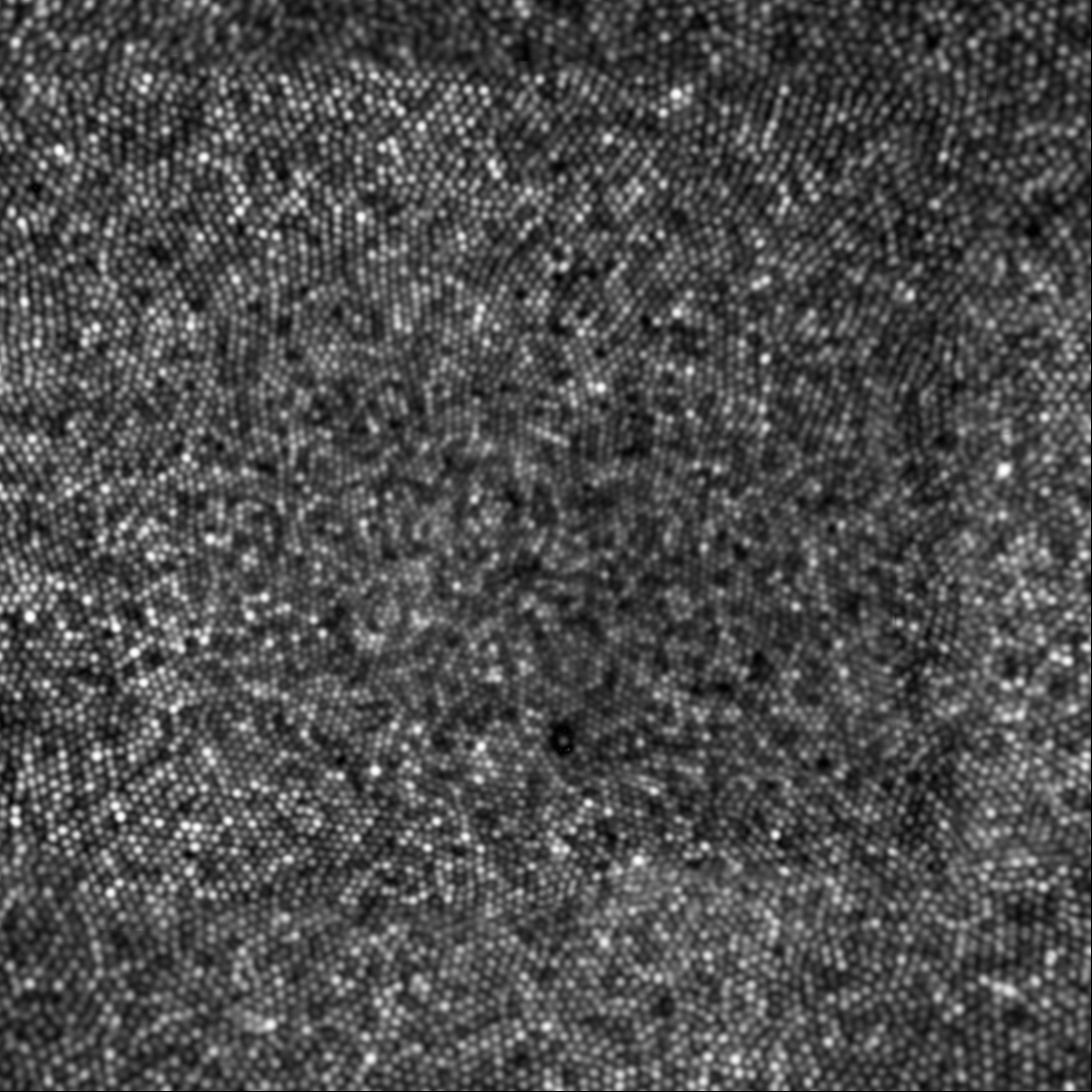

Supplement: Supplement 3 [file tvst-13-6-18_s003.zip › JC_11655_visit1_300um.tif]

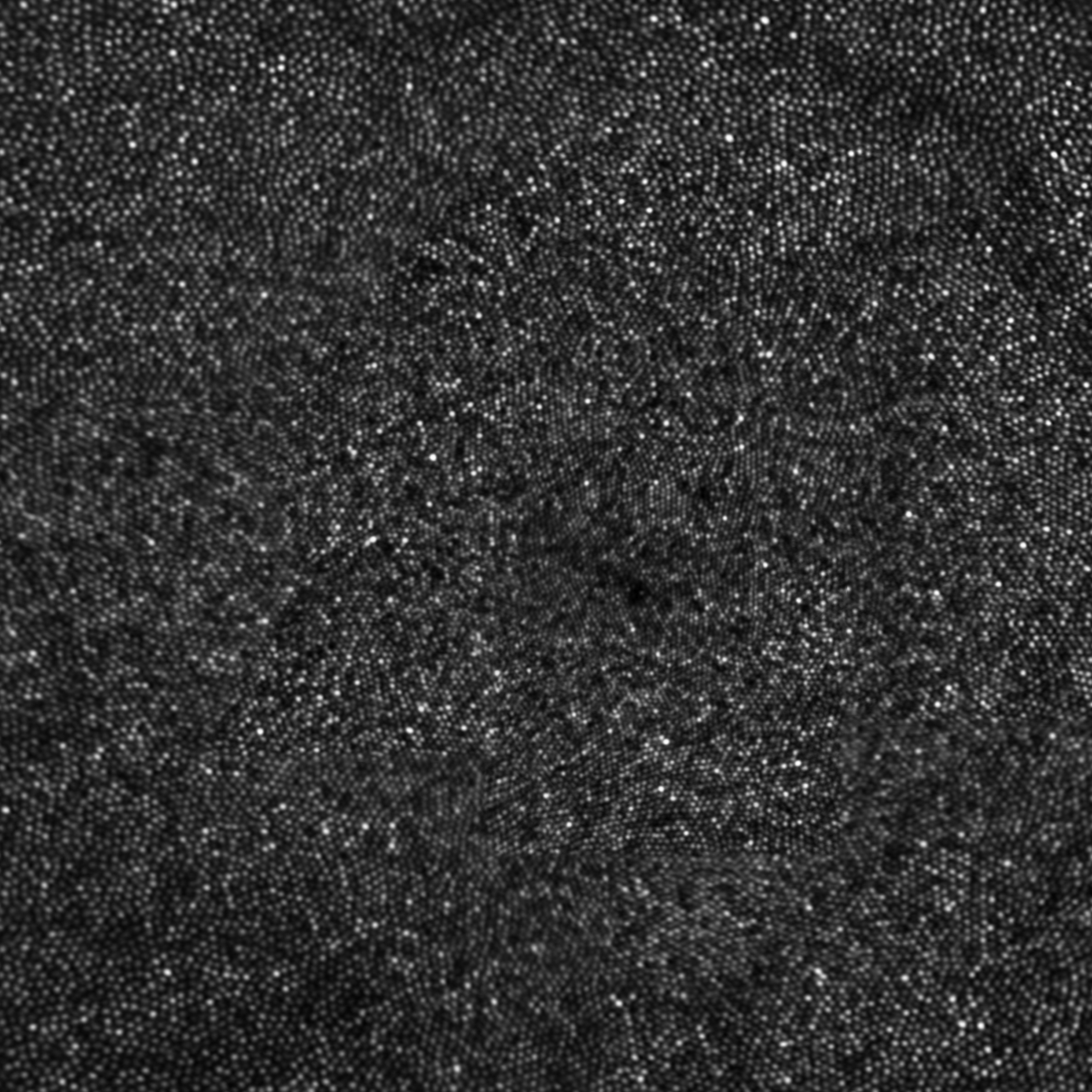

Supplement: Supplement 3 [file tvst-13-6-18_s003.zip › JC_11655_visit2_500um.tif]

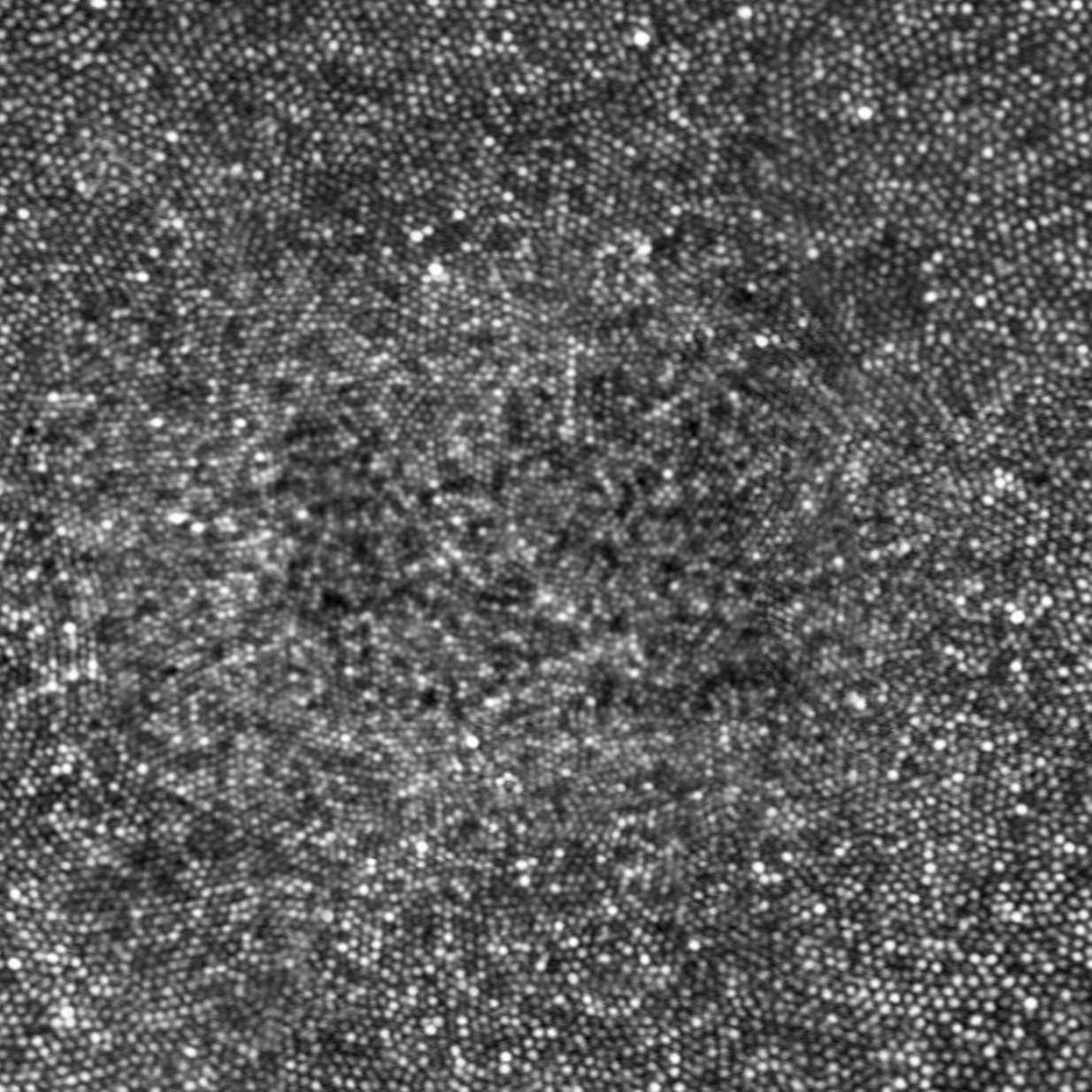

Supplement: Supplement 3 [file tvst-13-6-18_s003.zip › JC_11661_visit1_300um.tif]

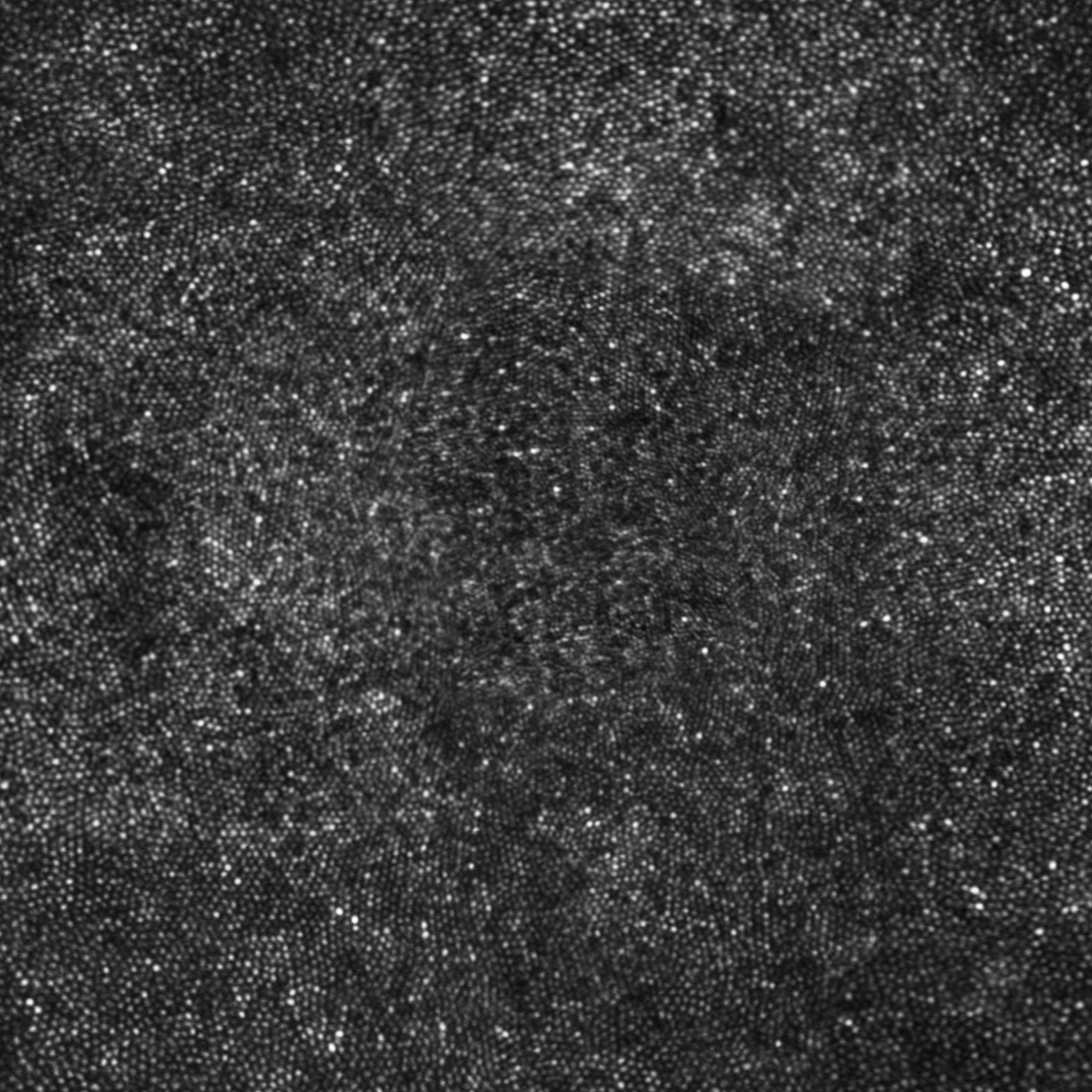

Supplement: Supplement 3 [file tvst-13-6-18_s003.zip › JC_11661_visit2_500um.tif]

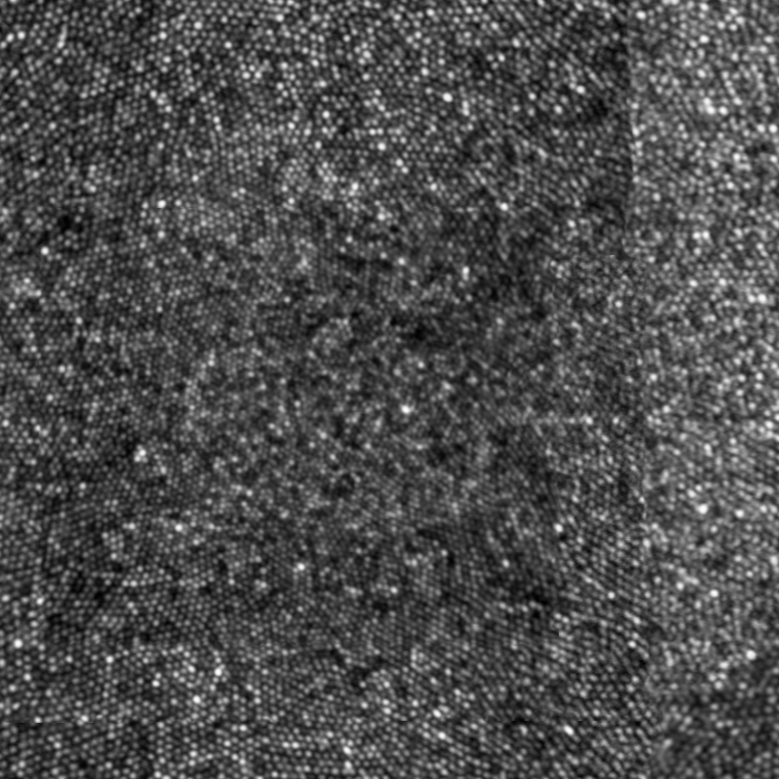

Supplement: Supplement 3 [file tvst-13-6-18_s003.zip › JC_12044_visit1_300um.tif]

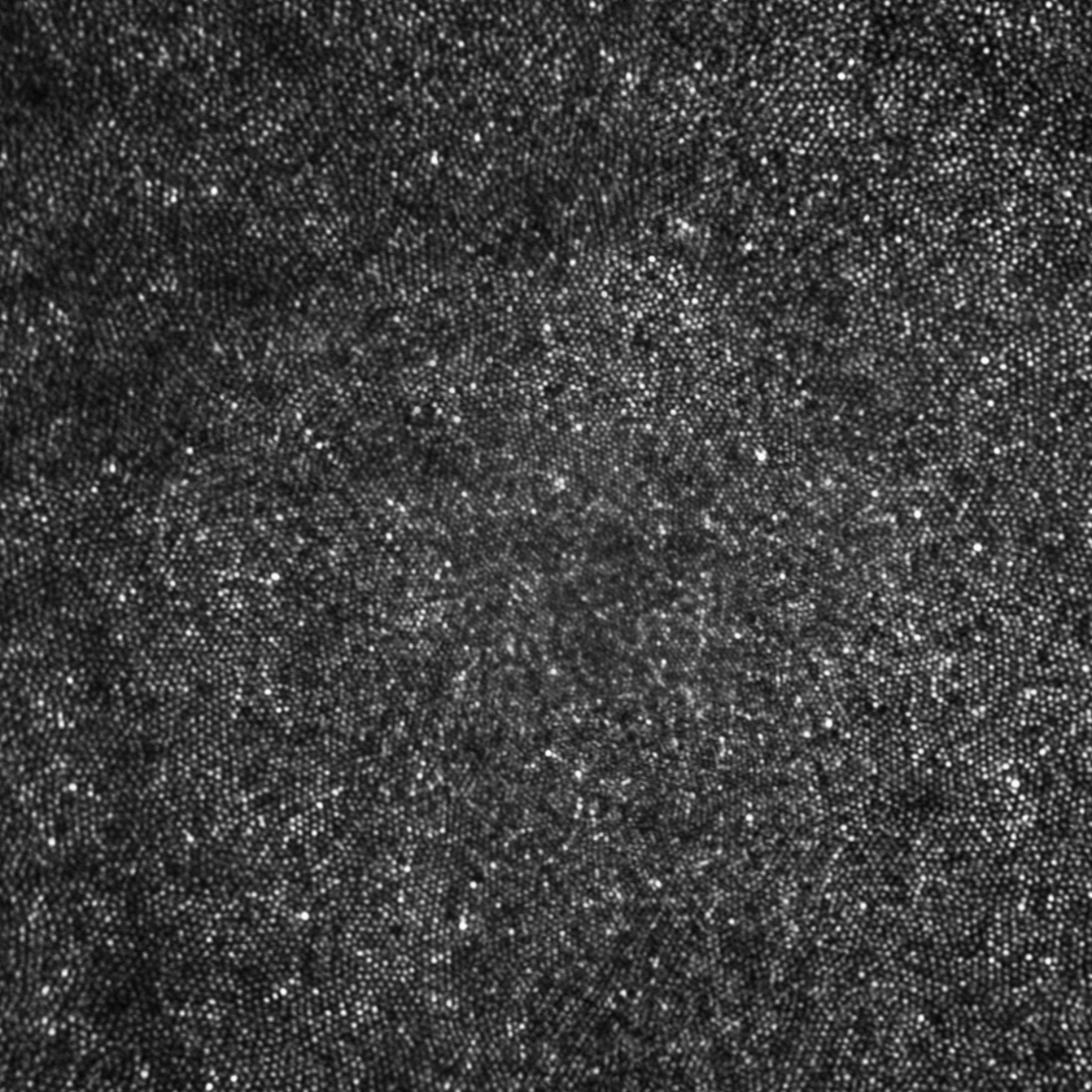

Supplement: Supplement 3 [file tvst-13-6-18_s003.zip › JC_12044_visit2_500um.tif]

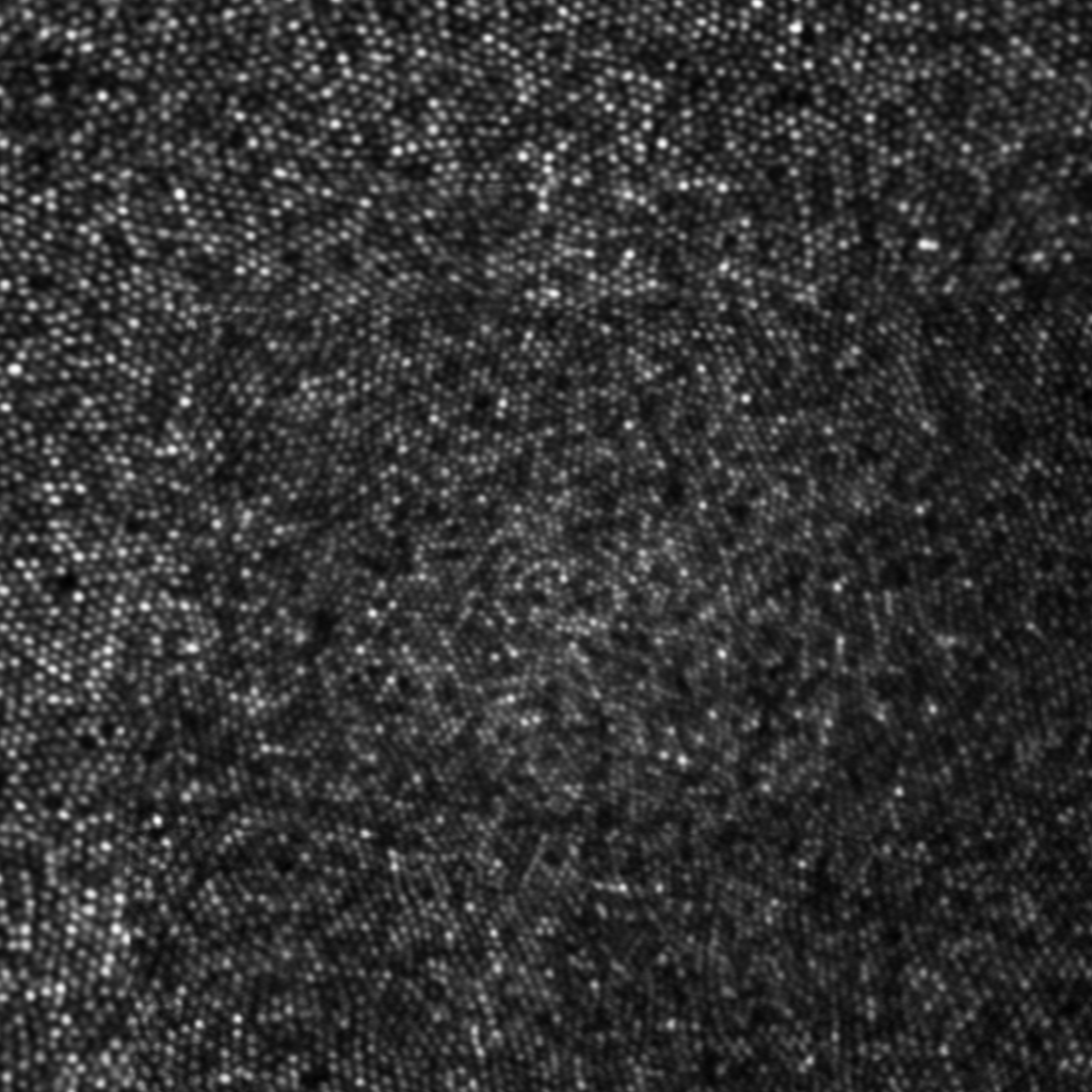

Supplement: Supplement 3 [file tvst-13-6-18_s003.zip › JC_12058_visit1_300um.tif]

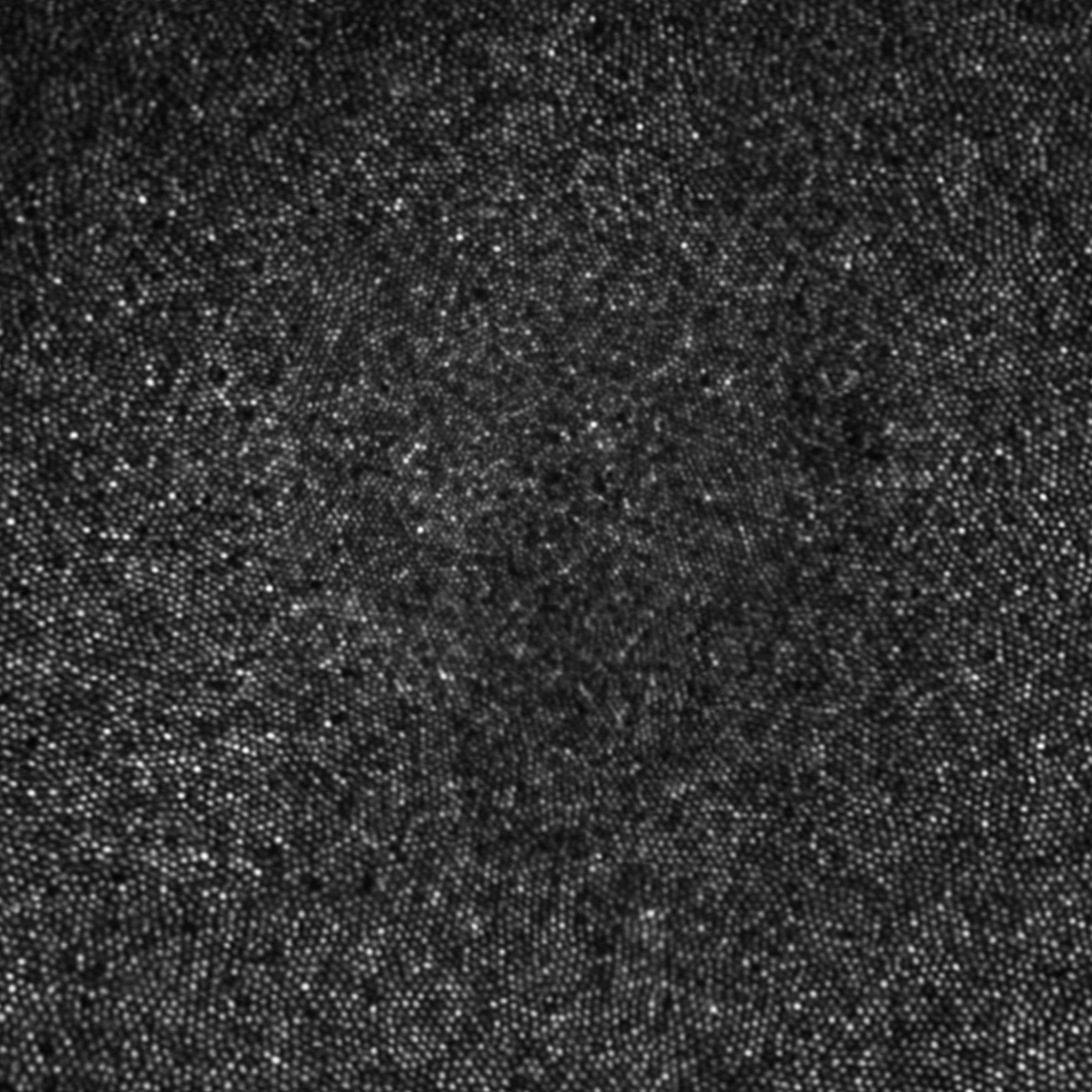

Supplement: Supplement 3 [file tvst-13-6-18_s003.zip › JC_12058_visit2_500um.tif]

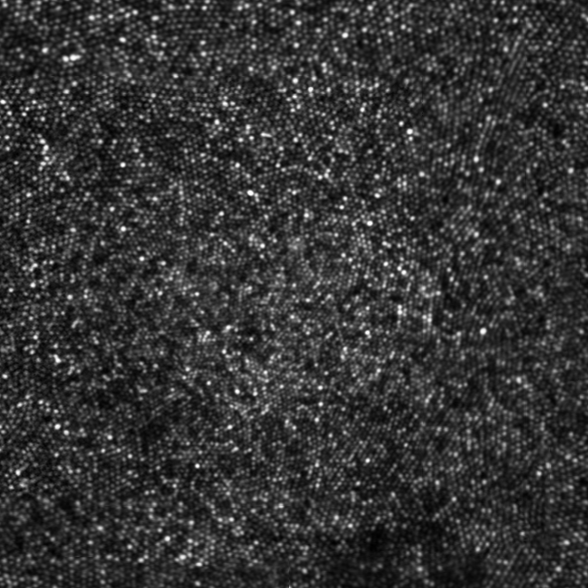

Supplement: Supplement 3 [file tvst-13-6-18_s003.zip › JC_12143_visit1_300um.tif]

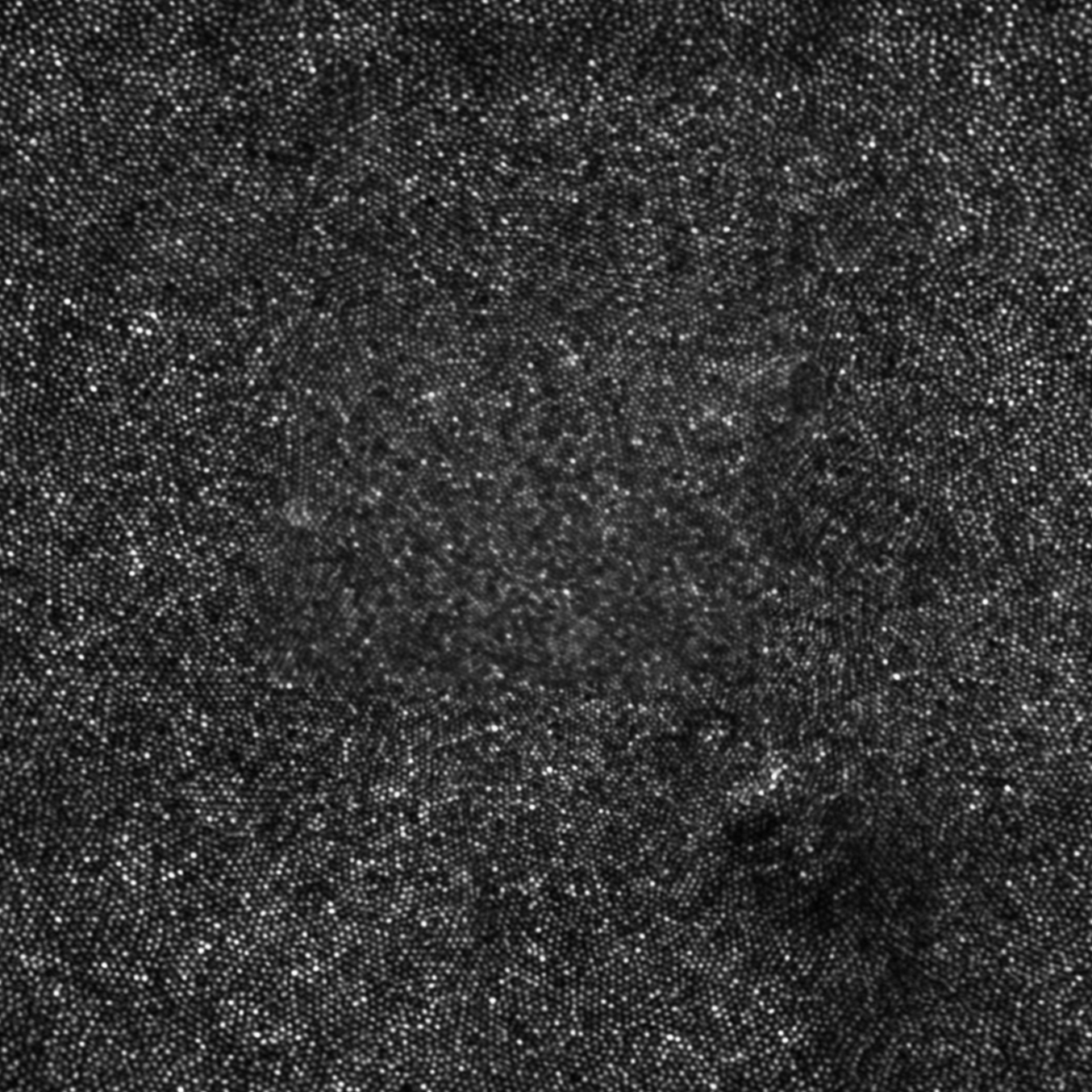

Supplement: Supplement 3 [file tvst-13-6-18_s003.zip › JC_12143_visit2_500um.tif]

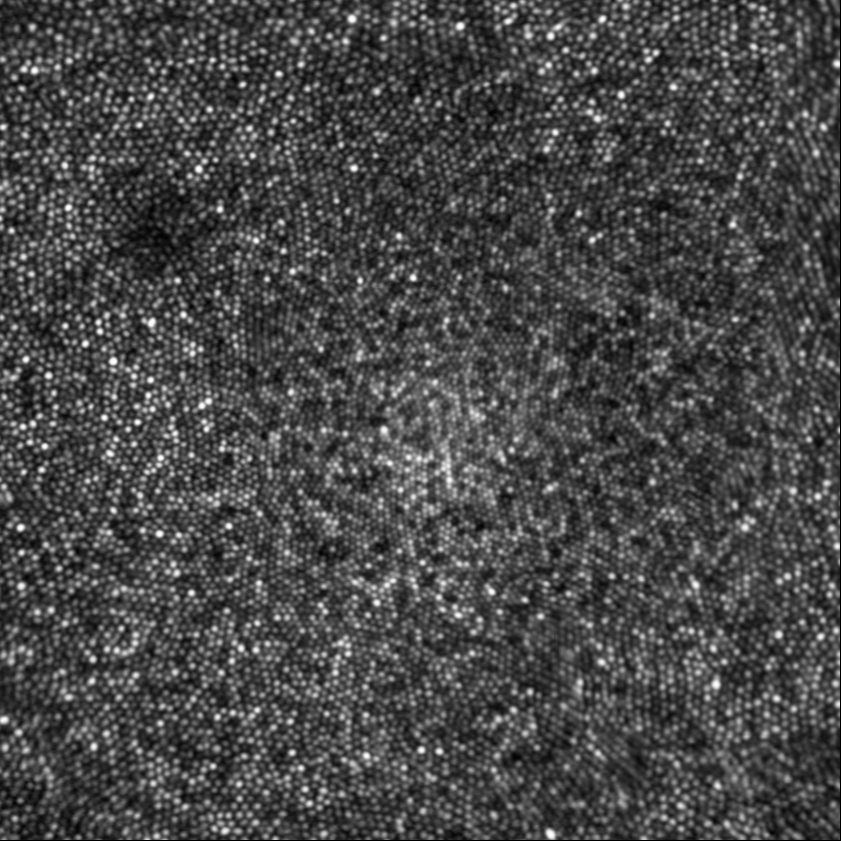

Supplement: Supplement 3 [file tvst-13-6-18_s003.zip › JC_12150_visit1_300um.tif]

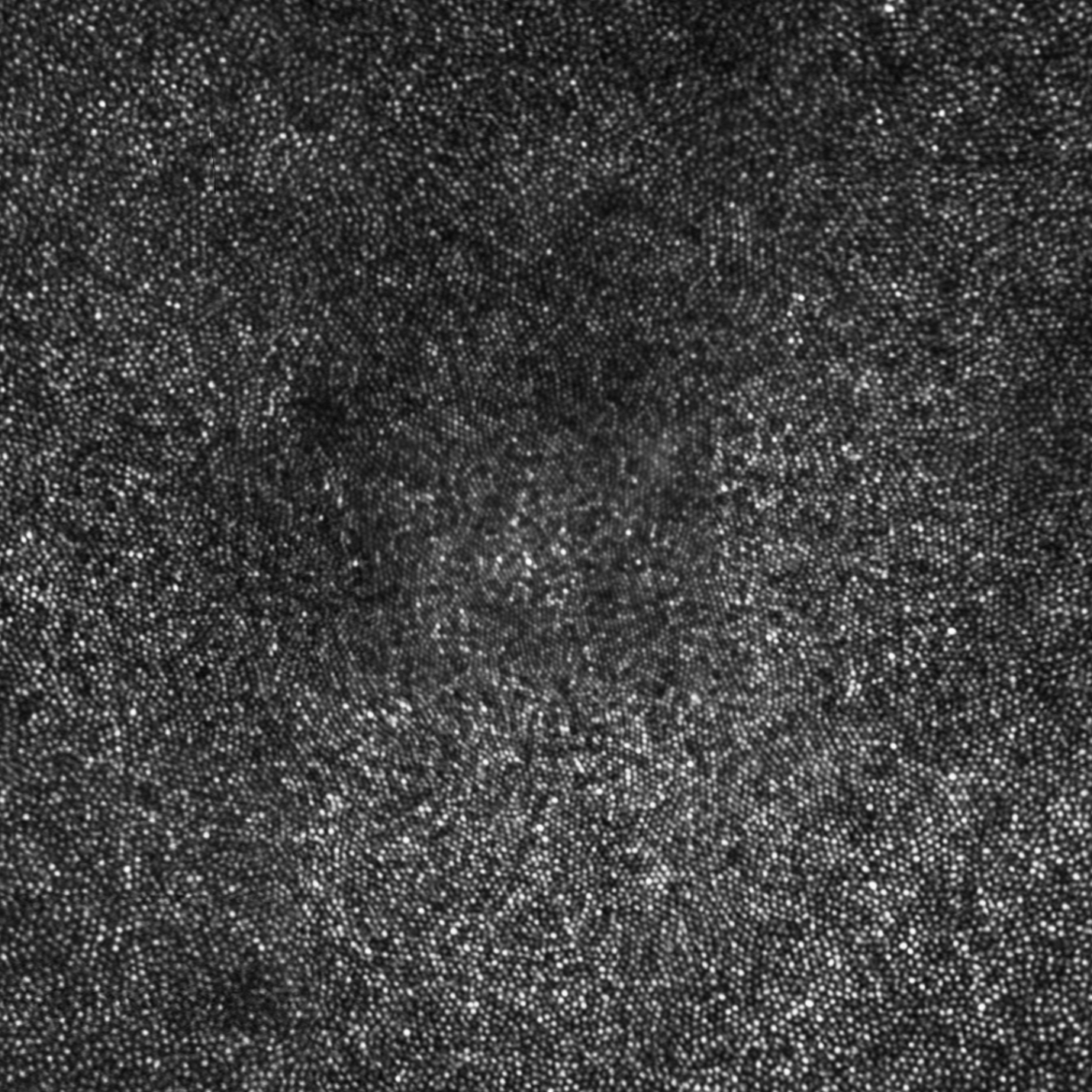

Supplement: Supplement 3 [file tvst-13-6-18_s003.zip › JC_12150_visit2_500um.tif]
